# miR-183-5p alleviates early injury after intracerebral hemorrhage by inhibiting heme oxygenase-1 expression

**DOI:** 10.18632/aging.103343

**Published:** 2020-06-29

**Authors:** Yu Wang, Yuejia Song, Yuxin Pang, Zihan Yu, Wei Hua, Yunhe Gu, Jiping Qi, He Wu

**Affiliations:** 1 Department of Pathology, First Clinical Hospital, Harbin Medical University, Harbin 150001, China; 2 Department of Endocrinology, First Clinical Hospital, Harbin Medical University, Harbin 150001, China

**Keywords:** microRNA-183-5p, heme oxygenase-1, inflammation, oxidative stress, intracerebral hemorrhage

## Abstract

Differences in microRNA (miRNA) expression after intracerebral hemorrhage (ICH) have been reported in human and animal models, and miRNAs are being investigated as a new treatment for inflammation and oxidative stress after ICH. In this study, we found that microRNA-183-5p expression was decreased in the mouse brain after ICH. To investigate the effect of miRNA-183-5p on injury and repair of brain tissue after ICH, saline, miRNA-183-5p agomir, or miRNA-183-5p antagomir were injected into the lateral ventricles of 8-week-old mice with collagenase-induced ICH. Three days after ICH, mice treated with exogenous miRNA-183-5p showed less brain edema, neurobehavioral defects, inflammation, oxidative stress, and ferrous deposition than control mice. In addition, by alternately treating mice with a heme oxygenase-1 (HO-1) inducer, a HO-1 inhibitor, a nuclear factor erythroid 2-related factor (Nrf2) activator, and Nrf2 knockout, we demonstrated an indirect, HO-1-dependent regulatory relationship between miRNA-183-5p and Nrf2. Our results indicate that miRNA-183-5p and HO-1 are promising therapeutic targets for controlling inflammation and oxidative damage after hemorrhagic stroke.

## INTRODUCTION

Intracerebral hemorrhage (ICH) has a high morbidity and mortality and accounts for 10%-15% of strokes [1]; it involves rupture of one or more blood vessels in the brain and blood leakage into the brain parenchyma [2, 3]. Brain injury caused by ICH occurs in two phases. The initial bleed disrupts the cellular architecture of the brain, and the hematoma increases intracranial pressure, impacting blood flow and leading to brain herniation [4]. The second phase of ICH injury lasts for hours or days and could be prevented [5, 6]. It involves a local inflammatory response [7] characterized by the release of clotting components (e.g., hemoglobin/iron) and perihematomal tissue damage (e.g., breakdown of the blood-brain barrier [BBB]) [8]. Evidence indicates that the release of thrombin, hemoglobin, and iron contributes to secondary injury [9–12]. Effective treatment is needed for the secondary injury caused by hemorrhagic stroke.

MicroRNAs (miRNAs) can induce posttranscriptional gene silencing, opening up a new strategy for treating human diseases [13]. Clinical and preclinical studies have confirmed that expression of a variety of miRNAs is altered in serum or cerebrospinal fluid after ICH [14–18]; these miRNAs are involved in BBB protection [19], the anti-inflammatory response [20, 21], inhibition of microglial activation [22] and neuronal apoptosis [19], and revascularization [23, 24].

In this study, we performed miRNA sequencing (miRNA-Seq) and bioinformatics analysis to identify miRNAs that affect the response to ICH. Among the 15 miRNAs with the most significant difference in expression after ICH, we observed a significant decrease in miR-183-5p. One putative target of miR-183-5p is heme oxygenase-1 (HO-1), a molecule widely reported to exacerbate ICH brain injury [25, 26]. Here, we investigated the effects and mechanism of action of miR-183-5p on injury and repair of brain tissue after ICH.

## RESULTS

### miRNA-183-5p expression in the brains of mice with ICH was significantly decreased

**Figure 1:**
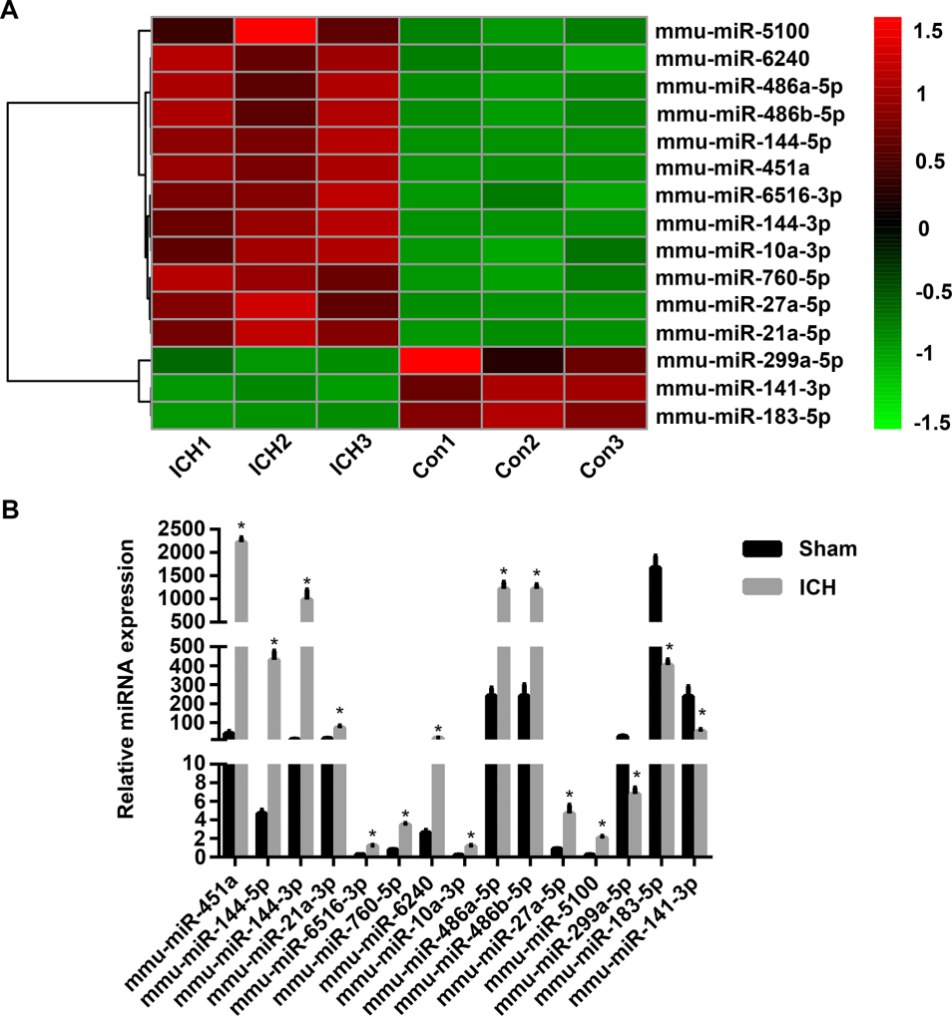
**The microRNA (miRNA) expression profiles of mouse brain tissue changed significantly after intracerebral hemorrhage (ICH).** (**A**) Heat map of 15 miRNAs with the most significant difference in expression after ICH. n = 3/group. (**B**) The expression levels of the top 15 miRNAs with the most significant difference in expression identified with sequencing were verified by qPCR. n = 8/group. Values are presented as the mean ± standard deviation. *P < 0.05 vs. the sham group. Con, control.

To identify miRNAs affecting early injury after ICH in mice, miRNA-Seq of the brain tissues of mice in the sham group (n = 3) and the ICH group (n = 3) was performed. Based on our previous study and other reports [26, 27], the observation time point of 3 days after ICH was chosen because this is the time of maximum activation of microglia, the main inflammatory cells responsible for brain injury after ICH. Compared with the sham group, the expression of 32 miRNAs was significantly increased in the ICH group, whereas that of 95 miRNAs was decreased. These differentially expressed miRNAs were found to affect multiple signaling pathways, including the Ras, MAPK, VEGF, and Toll-like receptor signaling pathways. miRNAs with a log_2_ fold change ≥ 2 and *P* < 0.05 were considered to have statistically significant differential expression. miRNAs with log_2_ fold change ≥ 2 were selected because significant differences in the expression of these miRNAs were found between the ICH group and the sham group and thus they were more likely to be involved in ICH injury. Thereafter, we ranked the differentially expressed miRNAs according to fold change in expression. The top 15 are displayed in Figure 1A. Thereafter, we performed quantitative polymerase chain reaction (qPCR) to verify these miRNAs and found that their changes in expression were consistent with the miRNA-Seq results (Figure 1B). Using miRanda software (omicX) to predict the targets of these 15 miRNAs, we found that one of the predicted targets of miRNA-183-5p was HO-1, which was confirmed in our previous study to be involved in early inflammation and oxidative stress injury after ICH. In addition, our analysis of the temporal expression patterns of miRNA-183-5p and HO-1 after ICH revealed a negative correlation (Supplementary Figure 1), suggesting that miRNA-183-5p regulates HO-1 expression.

### miR-183-5p reduced neurologic deficits, BBB permeability, and lesion volume after ICH

**Figure 2:**
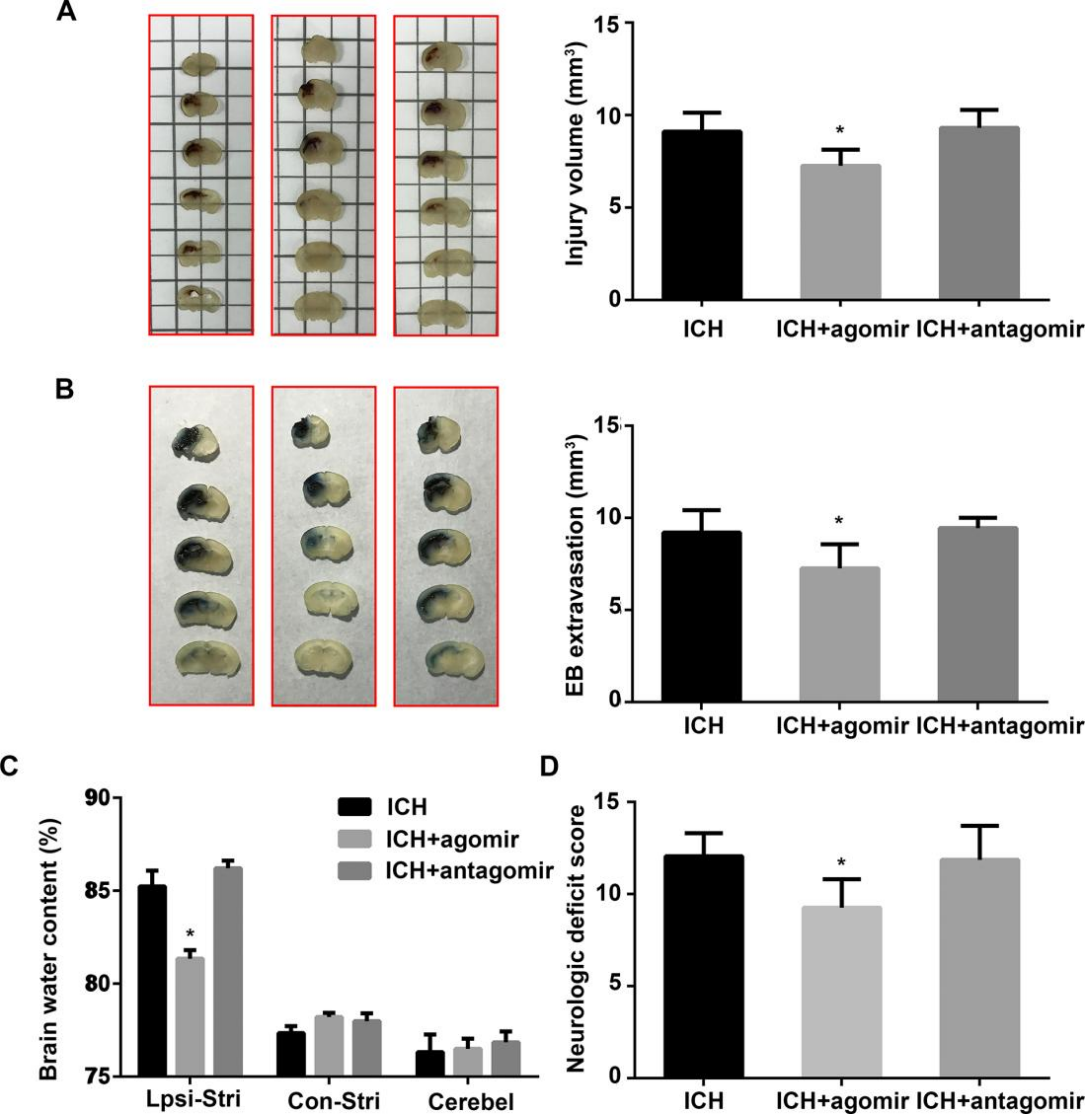
**Administration of miR-183-5p reduced neurologic deficits, blood-brain barrier permeability, and brain injury volume after intracerebral hemorrhaging (ICH).** (**A**) Left: representative images of a series of brain slices from different groups at 3 days after ICH. Right: quantitative analysis of hematoma volume. n = 8/group. (**B**) Left: representative images of brain slices from different groups at 3 days after ICH stained with Evans blue (EB). Right: quantitative analysis of EB extravasation. n = 8/group. (**C**) Brain water content in the different groups at 3 days after ICH. n = 8/group. Ipsi-Stri, ipsilateral striatum; Con-Stri, contralateral striatum; Cerebel, cerebellum. (**D**) Neurologic deficit scores of mice at 3 days after ICH. n = 24/group. Values are presented as the mean ± standard deviation. *P < 0.05 vs. the ICH group.

Lesion volumes were measured morphometrically (image analysis) 3 days after ICH. As shown in Figure 2A, lesions were smaller in the agomir group than in the control group (*P* < 0.05), and antagomir-treated mice exhibited larger lesions, although the difference in lesion volume was not significant (*P* > 0.05). BBB permeability was evaluated by Evans blue (EB) extravasation and found to be significantly decreased (*P* < 0.05) in the agomir group, but unchanged in the antagomir group, compared to the control group (Figure 2B). In addition, brain edema in the ipsilateral striatum was significantly decreased by agomir pretreatment (*P* < 0.05) but not by antagomir pretreatment (Figure 2C). Our results using miRNA agomir and antagomir revealed that miR-183-5p upregulation decreased neurologic deficits on day 3 (*P* < 0.05) (Figure 2D); in contrast, miR-183-5p downregulation increased neurologic deficits, although not significantly (*P* > 0.05) (Figure 2D).

### miR-183-5p alleviated early inflammation after ICH

**Figure 3:**
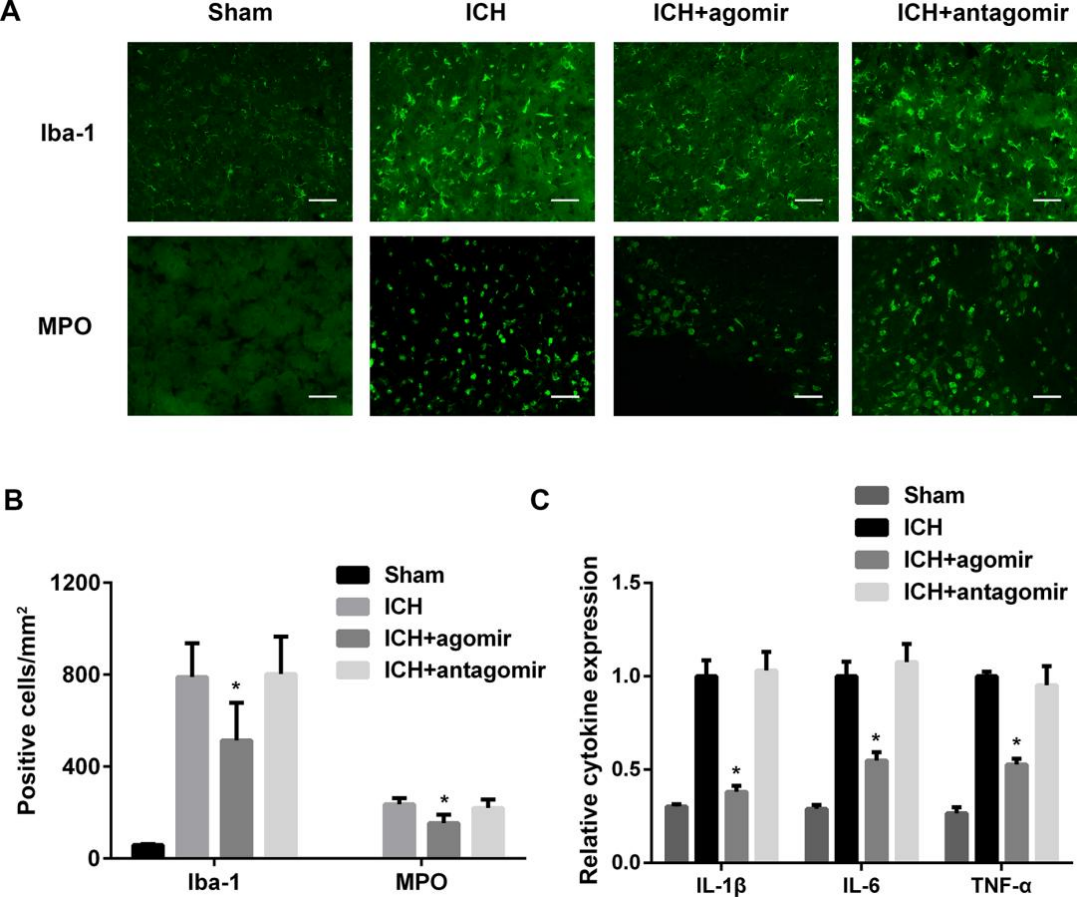
**Treatment with miR-183-5p alleviated early inflammation after intracerebral hemorrhage (ICH).** (**A**) Representative immunofluorescence images of Iba-1–positive microglia and myeloperoxidase (MPO)-positive neutrophils in different groups at 3 days after ICH. n = 8/group. (**B**) Quantitative analysis of Iba-1– or MPO-positive cells in (**A**). Scale bars = 50 μm. (**C**) Quantitative analysis of cytokine expression in the brains of mice from different groups at 3 days after ICH. n = 8/group. Values are presented as the mean ± standard deviation. *P < 0.05 vs. the ICH group.

To determine the effect of miR-183-5p on microglia activation and leukocyte infiltration after ICH, we performed an immunofluorescence experiment. We found that the number of activated microglia in the perihematomal area was lower in the agomir group (*P* < 0.05), but not the antagomir group (*P* > 0.05), when compared to the control group (Figure 3A and 3B). In addition, the number of MPO-positive neutrophils in the hemorrhagic striatum was significantly lower in the agomir group compared with the control group (Figure 3A and 3B).

The effect of miR-183-5p on inflammation was also studied in vivo. ELISA showed that the inflammatory factors IL-1β, IL-6, and TNF-α were decreased significantly in the agomir group (*P* < 0.05) and slightly, but not significantly, decreased in the antagomir group (*P* > 0.05) (Figure 3C). BV2 microglia cocultured with hemin and miRNA agomir or antagomir were used to study the response of inflammatory factors to miRNA regulation in vitro. Culture supernatants from BV2 microglia treated with agomir or antagomir were collected, and IL-1β, IL-6, and TNF-α levels were determined to be similar to the results obtained in vivo (Supplementary Figure 2).

### miR-183-5p alleviated oxidative damage after ICH

**Figure 4:**
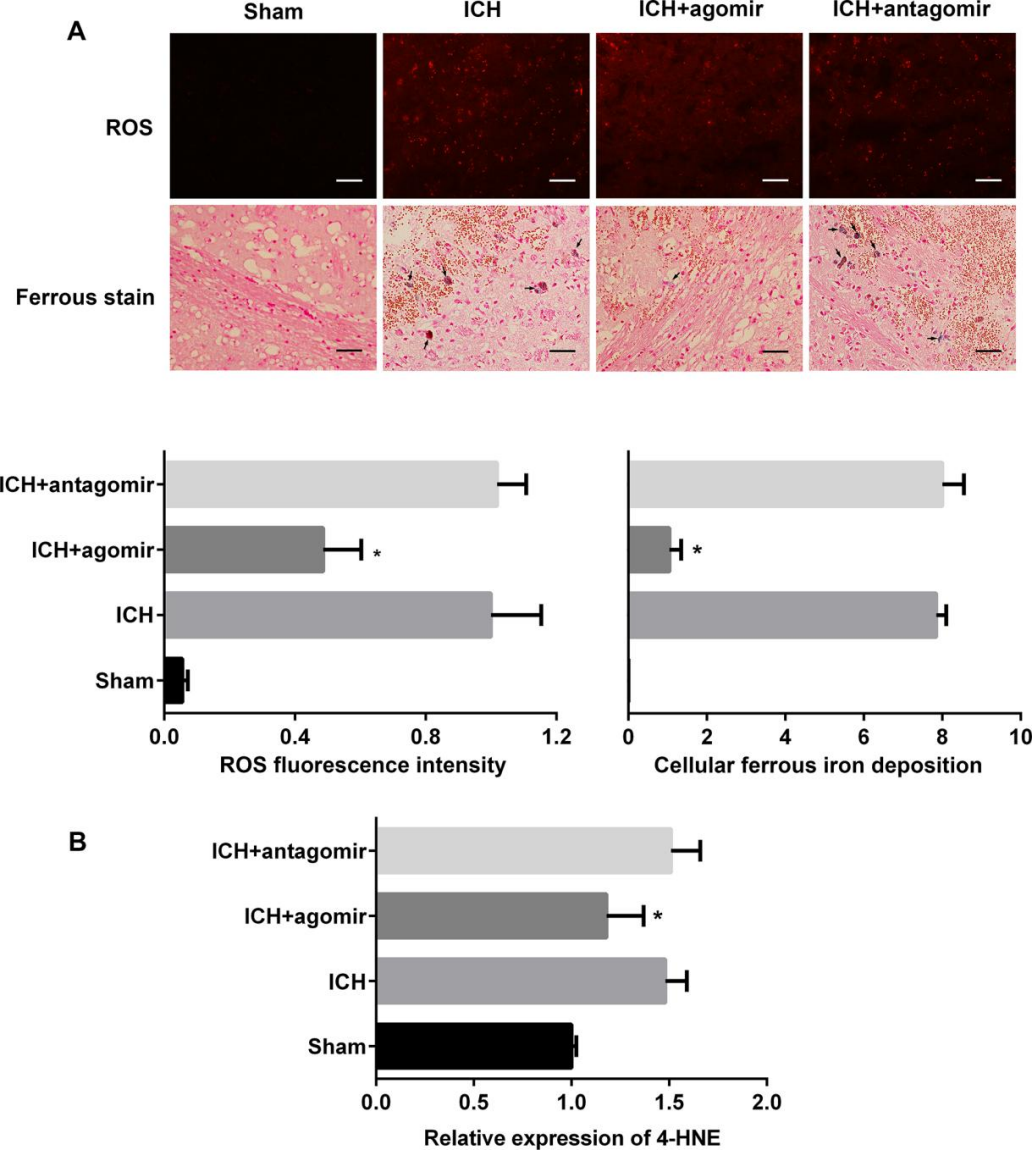
**Treatment with miR-183-5p alleviated oxidative damage after intracerebral hemorrhaging (ICH).** (**A**) Representative immunofluorescence images showing hydroethidine-positive reactive oxygen species (ROS) (n = 8/group) and ferrous deposition stained with Lillie dye (n = 8/group) in different groups at 3 days after ICH. Arrows indicate ferrous deposition in cells. Quantitative analysis of ROS fluorescence intensity and ferrous deposition in cells corresponding to the above are shown below. Scale bars = 50 μm. (**B**) Quantitative analysis of 4-HNE in the brains of mice from different groups at 3 days after ICH. n = 8/group. Values are presented as the mean ± standard deviation. *P < 0.05 vs. the ICH group.

We next determined whether miRNA-183-5p is involved in the regulation of reactive oxygen species (ROS) and the production of divalent iron. The fluorescent indicator hydroethidine was used to examine ROS production in situ (Figure 4A and Supplementary Figure 3A). In vivo, the fluorescence intensity of hydroethidine was significantly lower in the agomir group (*P* < 0.05) but nearly unchanged in the antagomir group (*P* > 0.05). The results of Lillie staining to determine ferrous deposition also showed that there were fewer positive cells in the agomir group (*P* < 0.05) (Figure 4A) but that there was no difference in positive cells between the ICH and antagomir groups (*P* > 0.05). Interestingly, the amount of ROS produced by individual BV2 microglia in vitro did not change significantly, but the number of viable microglia was significantly reduced (Supplementary Figure 3A). This finding was consistent with the trends observed in vivo, as shown by the miRNA-183-5p–mediated reduction in ROS production (Supplementary Figure 3B). The lipid peroxidation product, 4-HNE, was assessed by ELISA. The in vivo (Figure 4B) and in vitro (Supplementary Figure 3C) results showed that miRNA agomir treatment decreased the 4-HNE protein level (*P* < 0.05), whereas antagomir treatment did not notably change the expression of 4-HNE (*P* > 0.05).

### HO-1 is a direct downstream target of miR-183-5p

**Figure 5:**
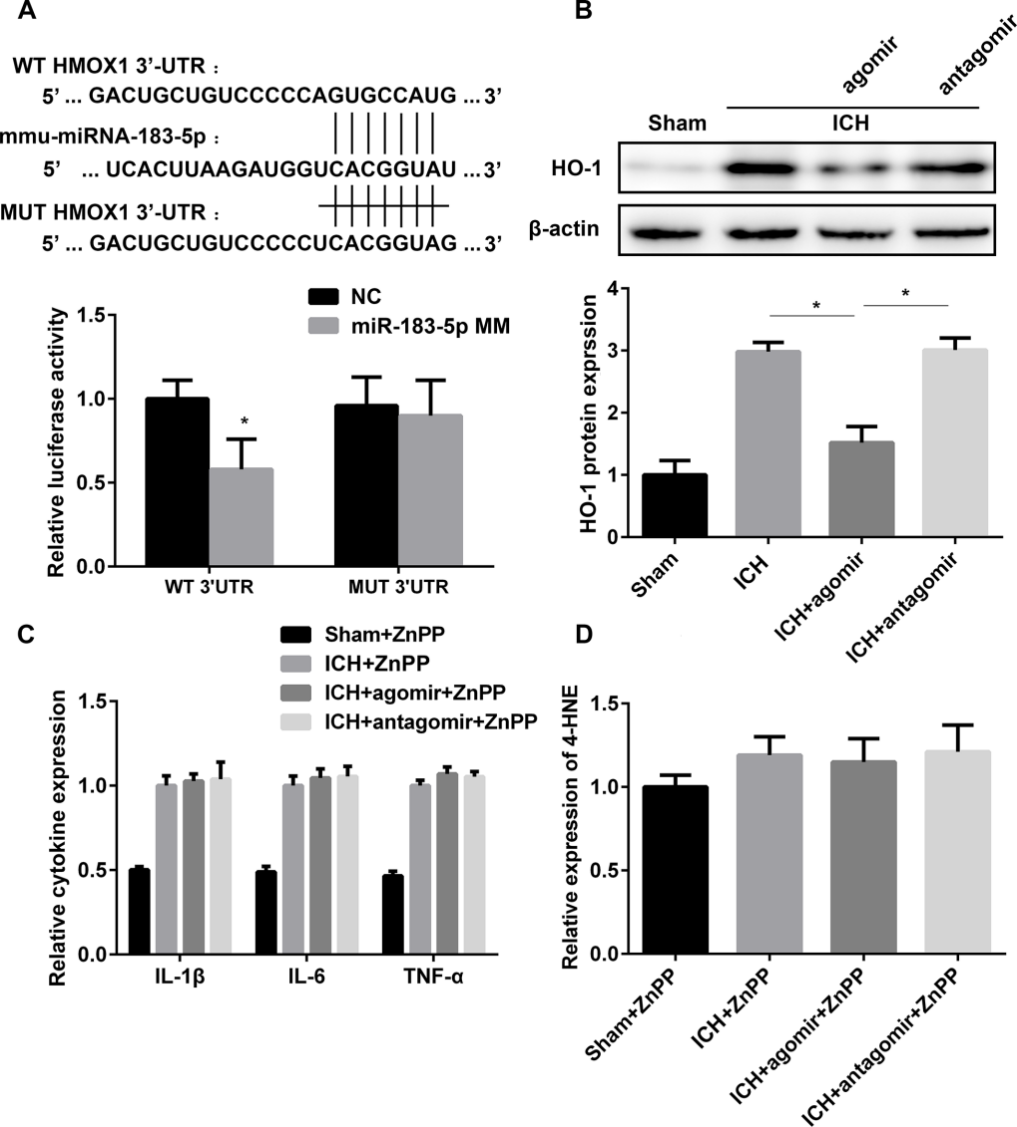
**miR-183-5p alleviated early inflammation and oxidative damage by directly targeting heme oxygenase-1 (HO-1).** (**A**) Above: schematic showing the potential miR-183-5p binding site in the HO-1 3’-untranslated region (3’-UTR). A mutant (MUT) HO-1 3’-UTR was introduced by replacing the wild type (WT) binding sequence with a mutant sequence. Below: inhibition of relative luciferase activity of HO-1 3’-UTR reporter molecules in human embryonic kidney 293 cells mediated by miR-183-5p. miR-183-5p MM, miR-183-5p mimic; NC, nontarget control. (**B**) Above: western blotting revealed that miRNA-183-5p downregulated HO-1 expression. Below: quantitative analysis of HO-1 protein expression in different groups. n = 8/group. (**C**) Quantitative analysis of cytokine expression in the brains of mice from different groups pretreated with the HO-1 inhibitor zinc protoporphyrin IX (ZnPP) at 3 days after ICH. n = 8/group. (**D**) Quantitative analysis of 4-HNE expression in the brains of mice from different groups pretreated with the HO-1 inhibitor ZnPP at 3 days after ICH. n = 8/group. Values are presented as the mean ± standard deviation. *P < 0.05 vs. the ICH group.

In view of the relationship between miRNA-183-5p and the time course of HO-1 expression (Supplementary Figure 1) and because HO-1 was predicted to be a target of miRNA-183-5p, we speculated that the regulatory function of miRNA-183-5p in early injury after ICH is mediated by downstream HO-1. A Dual-Luciferase Reporter Assay was performed to verify the relationship between miR-183-5p and HO-1 mRNA. Fragments of the HO-1 mRNA 3’-UTR containing either the binding site for miR-183-5p or a MUT binding site were designed (Figure 5A), and HEK293 cells were cultured for this purpose. Cotransfection of the wild-type (WT) 3’-UTR with miR-183-5p mimic significantly reduced the relative luciferase activity (*P* < 0.05), but cotransfection of the MUT 3’-UTR with miR-183-5p mimic did not (*P* > 0.05) (Figure 5A). Agomir and antagomir were administered in vivo via intracerebroventricular injection, immediately before collagenase injection. The expression of HO-1 in the agomir group decreased significantly (*P* < 0.05), but there was no significant difference in its expression between the antagomir group and the ICH group (*P* > 0.05, Figure 5B). In vitro, after incubation with 20 mM hemin for 1 hour, BV2 microglia were transfected with agomir or antagomir for 24 hours. HO-1 expression was decreased in the agomir group (*P* < 0.05) and was not significantly increased in the antagomir group (*P* > 0.05, Supplementary Figure 4A).

To further confirm whether miRNA-183-5p is involved in neuroinflammation and oxidative stress through HO-1, inflammatory factors and 4-HNE were measured in the miR-183-5p up- and downregulation groups after HO-1 inhibition by ZnPP. The in vitro and in vivo results indicated no difference in the expression of IL-1β, IL-6, TNF-α, or 4-HNE among the ICH, agomir, and antagomir groups (*P* > 0.05) (Figure 5C and 5D, Supplementary Figure 4B). This suggests that the protective effect of miRNA-183-5p on early injury in patients with ICH is achieved by inhibiting HO-1 expression.

### miRNA-183-5p and HO-1 affect microglial survival after ICH

**Figure 6:**
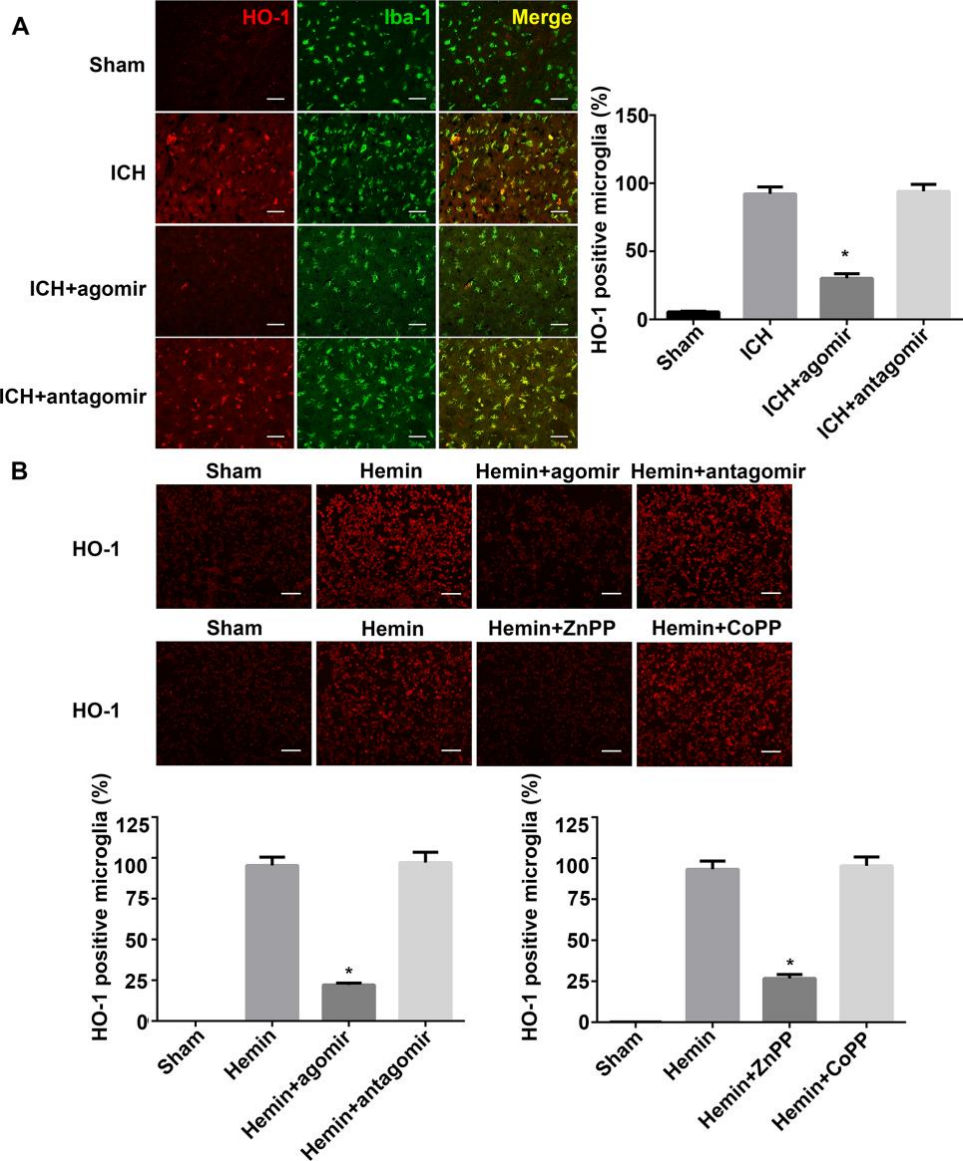
**miRNA-183-5p affected microglial survival by targeting heme oxygenase-1 (HO-1) after intracerebral hemorrhage (ICH).** (**A**) Left: representative immunofluorescence images of HO-1 in Iba-1–positive microglia at 3 days after ICH. Right: percentage of both Iba-1– and HO-1–positive cells in Iba-1–positive microglia. Scale bars = 50 μm, n = 8/group. *P < 0.05 vs. the ICH group. (**B**) Above: representative immunofluorescence images of HO-1 in BV2 microglia from different groups at 24 hours after hemin treatment. Below: percentage of HO-1–positive BV2 microglia. Scale bars = 50 μm, n = 3/group. *P < 0.05 vs. the hemin group. ZnPP, zinc protoporphyrin IX; CoPP, cobalt protoporphyrin IX.

Our previous study and other reports have demonstrated that HO-1 is expressed primarily in the microglia after ICH in mice. In addition, downregulation of HO-1 reduced the number of activated microglia [25, 26, 28]. Fluorescence microscopy analysis of HO-1 expression in this study indicated that, as previously reported, HO-1 was expressed mostly in the microglia after ICH and that HO-1 expression was significantly lower in the miRNA agomir group (*P* < 0.05, Figure 6A). Although HO-1 expression was slightly higher in the miRNA antagomir group (Figure 6A), this difference was not statistically significant (*P* > 0.05). The in vitro expression of HO-1 revealed that the regulatory effect of miRNA-183-5p was similar to that of HO-1 activator or inhibitor (Figure 6B).

In addition, exogenous miRNA-183-5p supplementation reduced the number of Iba-1–positive microglia. The downregulation of HO-1 by miRNA-183-5p had a damaging effect on microglia in the presence of hemin. The in vitro CCK-8 assay showed that the viability of microglia in the hemin+agomir group was significantly decreased, whereas the viability in the hemin+antagomir group was essentially the same as that in the hemin-alone group (Supplementary Figure 5). In vitro experiments revealed that increasing the expression of HO-1 protected microglia in the presence of hemin. Thus, miR-183-5p may decrease microglia survival and inhibit microglia from promoting inflammation and oxidative damage.

### MiR-18 3-5p regulates HO-1 independent of Nrf2

Because Nrf2 is recognized as the main regulator of HO-1 [29, 30], we determined the relationship between miR-183-5p, HO-1, and Nrf2 after ICH. After ICH, Nrf2^-/-^ mice exhibited low HO-1 expression, and treatment with tBHQ, an Nrf2 activator, increased HO-1 expression in WT mice with ICH (Supplementary Figure 6A). When HO-1 expression was regulated by CoPP or ZnPP, p-Nrf2 was increased or decreased, respectively (Supplementary Figure 6B). In miR-183-5p agomir–treated Nrf2^-/-^ mice, HO-1 expression was significantly inhibited (*P* < 0.05), but in miR-183-5p antagomir–treated Nrf2^-/-^ mice, HO-1 expression was slightly, although not significantly, increased (*P* > 0.05) (Supplementary Figure 6C). Among the Nrf2^-/-^ mice, miR-183-5p expression was higher in the group pretreated with the HO-1 inhibitor ZnPP (*P* < 0.05) and slightly reduced in the group pretreated with the HO-1 activator CoPP (*P* > 0.05, Supplementary Figure 6D).

**Figure 7:**
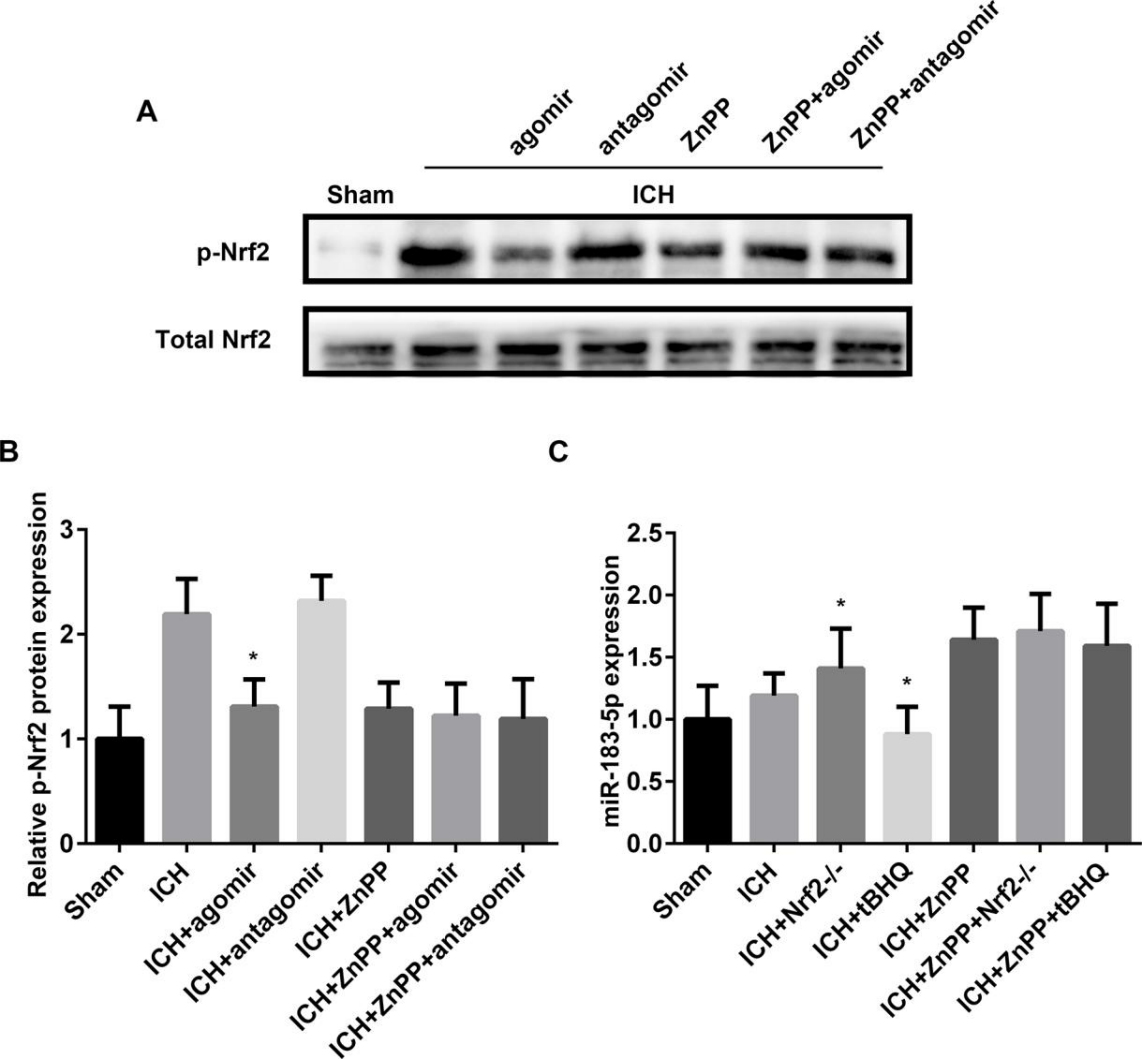
**miR-183-5p regulated heme oxygenase-1 (HO-1) independent of Nrf2.** (**A**) Western blotting revealed that miRNA-183-5p is an HO-1–dependent inhibitor of Nrf2 phosphorylation. n = 8/group. (**B**) Quantitative analysis of the relative expression of p-Nrf2 protein in (**A**). (**C**) Quantitative analysis of the HO-1–dependent inhibitory effect of Nrf2 on miR-183-5p by RT-qPCR. n = 8/group. Values are presented as the mean ± standard deviation. *P < 0.05 vs. the intracerebral hemorrhaging (ICH) group. ZnPP, zinc protoporphyrin IX; tBHQ, tert-butylhydroquinone.

Next, we sought to verify whether miRNA-183-5p could directly affect the function of Nrf2 in the ICH model. Previous studies have shown that phosphorylation of the Neh2 domain of Nrf2 at Ser-40 promotes the dissociation of Nrf2 from Keap1 and its translocation into the nucleus, where it induces the activation of antioxidant response elements (AREs) [31, 32]. tBHQ has been shown to promote the phosphorylation of Nrf2 [33]. p-Nrf2 expression, measured by western blotting, indicated that miR-183-5p upregulation suppressed the phosphorylation of Nrf2 (*P* < 0.05), whereas this regulatory effect of miR-183-5p was absent in the absence of HO-1 (*P* > 0.05, Figure 7A and 7B). Furthermore, we determined whether activation of Nrf2 directly affected the expression of miRNA-183-5p. The reverse transcriptase (RT) qPCR results confirmed that activation or knockout of Nrf2 suppressed or promoted miR-183-5p expression (*P* < 0.05), respectively, but that this regulatory effect of Nrf2 was also absent in the absence of HO-1 (*P* > 0.05, Figure 7C).

## DISCUSSION

In this study, we observed changes in the expression of many miRNAs in the brain tissue of mice after ICH. Among these miRNAs, miR-183-5p was demonstrated to have a protective effect on ICH mice. After injecting agomir-183-5p into the lateral ventricles of ICH mice, we observed a decrease in iron accumulation, brain ROS production, BBB injury, the inflammatory response, neurologic impairment, and microglial activation. Although previous studies have shown that the HO-1 activator CoPP can significantly increase the expression of HO-1, the antagomir of miR-183-5p did not upregulate the expression of HO-1 as expected. We have found that the physiologic level of miR-183-5p is not high in brain tissue. Agomir is a chemically modified miRNA; in vivo injection of agomir can greatly increase the expression of miR-183-5p and thus promote its role in inhibiting HO-1. Antagomir can combine with miR-183-5p after ICH, resulting in the loss of its ability to bind HO-1 mRNA. However, due to the further decrease in miR-183-5p expression after ICH, the degree of miR-183-5p inhibition by antagomir is not enough to significantly restore the effect of HO-1.

Free heme is harmful to cells, and the metabolization of heme into ferrous iron (Fe^2+^) prevents it from producing an excess of ROS [34]. In this regard, HO-1, as a metabolic enzyme of heme, has a protective effect on brain parenchyma in the event of a hemorrhage. However, we observed that inflammation and oxidative stress increased when HO-1 increased in mouse brain tissue. It is worth noting that during ICH, the types of cells enriched by HO-1 are primarily microglia. This suggests that the role of HO-1 in promoting injury during ICH may be related to this uneven cell distribution. Microglia, the intrinsic immune cells in the central nervous system, have been reported to respond rapidly to injury after ICH [6, 27, 35], contributing to inflammation and oxidative stress [1, 28, 36, 37]. It is possible that high HO-1 expression protects microglia in the ICH environment [38], further promoting inflammation and oxidative damage. In our in vitro experiment, we used CCK-8 to determine the survival rate of BV2 microglia treated with hemin. We found that, compared with the control group, HO-1 expression and the survival rate of BV2 cells treated with miR-183-5p agomir decreased and that the level of inflammatory factors secreted into the cell suspension decreased in conjunction with the number of cells. Thus, although HO-1 has a protective effect on microglia, the increase in the number of surviving microglia compared to other cells promotes inflammation and oxidative damage in the brain after ICH.

Iron also plays an important role in oxidative stress after ICH [39, 40]. Many studies have confirmed that bivalent iron promotes production of ROS in cerebral hemorrhage models; in addition, removal of divalent iron reduces brain injury [40–42]. Normally, HO-1 promotes decomposition of heme, releasing Fe^2+^, which can be degraded. However, in the acute phase of ICH, increased heme metabolism produces Fe^2+^ in amounts that exceed the metabolic capacity of the brain. Subsequently, the release of Fe^2+^ triggers the Fenton reaction [40], prompting microglia to produce large amounts of ROS. In our current study, the levels of Fe^2+^ and ROS in the brain tissue of the ICH mice receiving agomir-183-5p were lower than in the brain tissue of the control group [26]. The products of heme degradation also produce carbon monoxide (CO) and biliverdin, both of which are protective against oxidative damage from an ROS attack [43, 44]. On the one hand, microglia degrade heme to avoid continuous oxidation; on the other hand, CO and biliverdin protect microglia from ROS. Heme, Fe^2+^, and inflammation promote early injury of brain tissue after ICH. Overactive microglia reduce the level of heme but rapidly increase Fe^2+^ and inflammation. In a previous study [26], we found that although HO-1 promotes brain injury in the early stages of ICH, it promotes the recovery of neurologic function in the later stages. This is related to the role of HO-1 in promoting the metabolism of heme and reducing continuous oxidation.

The putative targets of miR-183-5p were predicted using miRanda software in this study (Supplementary Table 1). We also determined the differentially expressed mRNAs (Supplementary Table 1) in the brain of mice 3 days after ICH by mRNA-Seq. These mRNAs (Supplementary Table 1), which are present not only in the predictive targets of miRNA-183-5p, but also in the differentially expressed mRNAs of brain tissues after ICH, may be the ones that competitively bind miR-183-5p and participate in the process of ICH. For example, it has been reported that neurons are protected by restoring HK2-mediated glucose uptake during ischemic brain injury [45]. Melatonin protects brain tissue by inhibiting Serpina3n-mediated neuroinflammation [46]. In addition, the brain tissue damage caused by IL-6 depends on the trans-signaling mechanism mediated by Serpina3n [47]. Lpxn [48], Sucnr1 [49], and Csf2rb2 [50] seem to be related to the migration of macrophages. Ptbp3 [51] can promote the growth and metastasis of colorectal cancer through the activation of HIF-1α, and HIF-1α is involved in the ICH process [52]. After ICH, these mRNAs may compete with HO-1 mRNA to combine miR-183-5p and may affect the regulation of ICH processes by miR-183-5p. To confirm the effect of miR-183-5p on ICH through HO-1, ZnPP was administered to downregulate HO-1 expression. As expected, when HO-1 was inhibited, there was no difference in the expression of inflammatory factors or 4-HNE between the agomir-treated, antagomir-treated, and ZnPP-treated ICH groups or in BV2 cells in vitro. These results suggest that miR-183-5p exerts its anti-inflammatory and antioxidant effects by inhibiting HO-1. We also observed that miR-183-5p and miR-6958-3p were the only miRNAs that targeted HO-1 after ICH. However, the difference in miR-6958-3p expression before and after ICH did not meet the log_2_ fold change ≥ 2 criterion, indicating that its participation in the ICH process is not as significant as that of miR-183-5p.

Nrf2 is a transcription factor that promotes upregulation of ARE-mediated antioxidant gene expression [53, 54]. Nrf2 is activated by cellular oxidative stress and electrophiles, and it upregulates many genes that decrease oxidative stress (e.g., superoxide dismutase and HO-1) or induce phase II metabolism (in which exogenous electrophiles, e.g., xenobiotics, are catabolized) [55]. In the current study, we found that HO-1 expression was greatly decreased in Nrf2^-/-^ ICH mice, whereas the Nrf2 activator, tBHQ, increased HO-1 expression, consistent with a report by Zhao et al. [56]. Although the experimental results from the ICH model of Nrf2 knockout mice confirmed the brain-protective effect of Nrf2 [57], HO-1 seems to weaken this effect as a by-product. Therefore, we wanted to determine whether exogenous miR-183-5p could reduce HO-1 expression without affecting the activation of Nrf2 or whether there is a feedback regulation mechanism between miR-183-5p and Nrf2. This information is important to determine whether miR-183-5p can be used to inhibit HO-1 and enhance the brain-protective effect of Nrf2 in the treatment of secondary injury after ICH.

We analyzed Nrf2 phosphorylation after up- and downregulation of miR-183-5p expression and found a negative correlation between p-Nrf2 and miR-183-5p expression. When HO-1 was knocked down, the regulation of miRNA-183-5p did not change the expression level of p-Nrf2. When phosphorylation of Nrf2 was inhibited, or promoted in Nrf2^-/-^ and tBHQ-treated mice, miRNA-183-5p was upregulated or downregulated, respectively; however, this regulatory relationship disappeared when HO-1 was knocked down. Therefore, the regulation of Nrf2 and miR-183-5p is related via HO-1.

We also found that exogenous miR-183-5p agomir is protective against ICH injury, and this effect is HO-1 dependent, similar to the effect of the HO-1 inhibitor ZnPP (Supplementary Figure 7) in our previous study [26]. The secondary injury of ICH is mainly manifested by clot-derived cytotoxic factors and neuroinflammation [58]. Hemoglobin from the clot induces NO production [59]. Hemoglobin also increases the permeability of the BBB by activating protease-activated receptor-1 (PAR-1) [60]. BBB destruction is the main cause of increased hematoma and brain edema after ICH [61]. Counterintuitively, HO-1, an enzyme that promotes hemoglobin metabolism, aggravates brain damage in the early stages of ICH. This may be due to the fact that hemoglobin and heme also directly activate microglia and that the IL-1β and TNF-α secreted by microglia increase the permeability of the BBB and the volume of hematoma [62]. In addition, activated microglia can also secrete chemokine to recruit neutrophils and monocytes in the circulation, further aggravating neuroinflammation [63–65]. One of the by-products of heme metabolism catalyzed by HO-1 is the increase of divalent iron, which increases the production of ROS and promotes oxidative damage.

To sum up, overexpression of miR-183-5p protects brain tissue and improves neurologic function after ICH by inhibiting HO-1 expression.

In conclusion, we have illustrated that miRNA-183-5p expression is significantly decreased after ICH in mice. Exogenous agomir-183-5p reduces oxidative stress and neuroinflammation by inhibiting HO-1 mRNA expression. In addition, we also confirmed that miR-183-5p is associated with Nrf2, but only indirectly, through HO-1. The miR-183-5p/HO-1 axis may be a novel therapeutic target for reduction of secondary neuroinflammation and oxidative damage in hemorrhagic stroke.

## MATERIALS AND METHODS

### Animals and experimental design

**Figure 8:**
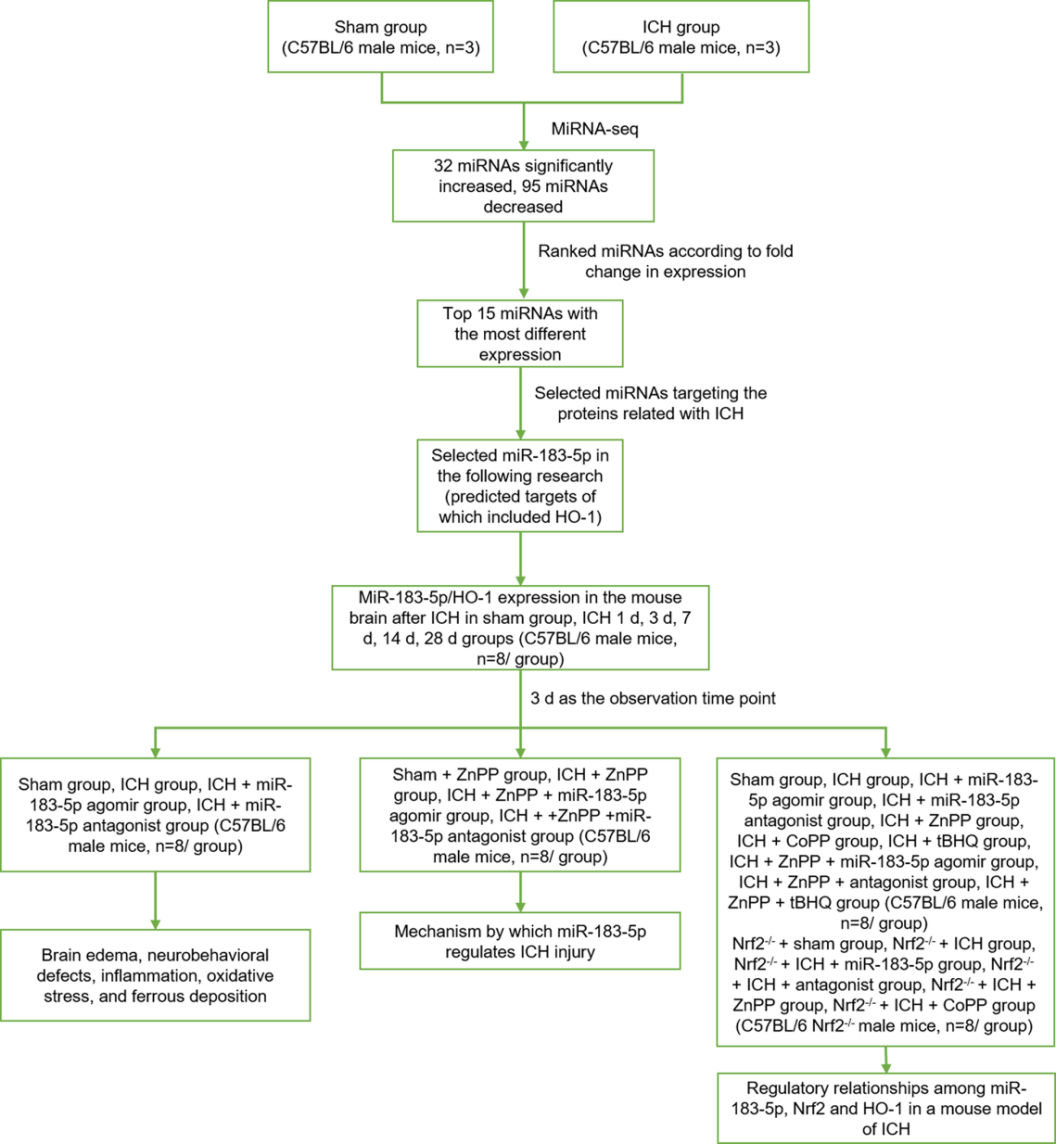
**The experimental design.** Sham group, sham operation group; ICH group, collagenase-induced intracerebral hemorrhage group; miRNA-seq, miRNA sequencing; miR-183-5p, microRNA-183-5p; HO-1, heme oxygenase-1; ICH 1 d, 3 d, 7 d, 14 d, 28 d groups, 1 day, 3 days, 7 days, 14 days, 28 days after collagenase-induced intracerebral hemorrhage groups; ZnPP, HO-1 inhibitor zinc protoporphyrin IX; Nrf2^-/-^, nuclear factor erythroid 2-related factor knockout; CoPP, HO-1 inducer cobalt protoporphyrin IX; tBHQ, Nrf2 activator tert-butylhydroquinone.

All animal experiments were approved by the Institutional Animal Care and Use Committee of Harbin Medical University, Harbin, China. Male C57BL/6 mice weighing 20-25 g (8 weeks old) were purchased from the Laboratory Animal Center of Harbin Medical University. Male Nrf2^-/-^ mice weighing 20-25 g (8 weeks old; CCME Dock No. CCME868348, Johns Hopkins University School of Medicine) were generously donated by Prof. Wang. The experimental design is shown in Figure 8.

Agomir-183-5p (miR40000212-4-5) and antagomir-183-5p (miR30000212-4-5) were synthesized by RiboBio (Guangzhou, China). The HO-1 inhibitor, zinc protoporphyrin IX (ZnPP, 282820), and inducer, cobalt protoporphyrin IX (CoPP, C1900), were purchased from Sigma-Aldrich (Sigma-Aldrich, St. Louis, MO, USA). Nrf2 activator tBHQ (8.41424) was also purchased from Sigma-Aldrich.

### ICH models

Collagenase was used to induce ICH, as described previously [41, 66, 67]. Briefly, mice were anesthetized with ketamine (100 mg/kg) and xylazine (10 mg/kg, intraperitoneal [IP] injection) and placed in a prone position on a stereotaxic apparatus (Zhongshi Dichuang, Beijing, China). An incision was made in the middle of the scalp, and a burr hole was drilled with a dental drill. Thereafter, a Hamilton syringe (Gaoge, Shanghai, China) was inserted stereotaxically through the hole into the right striatum (coordinates [68]: 0.8 mm anterior and 2.2 mm lateral of the bregma, 3.0 mm deep). ICH was induced by administering 0.4 μL of collagenase VII-S (0.075 U in 500 nL of saline, Sigma-Aldrich) over a 5-minute period. To avoid backflow, the microsyringe was kept in situ for a further 10 minutes before being slowly withdrawn. Following collagenase infusion, the craniotomies were sealed with bone wax, and the wounds were sutured. Sham operations were performed via stereotaxic injection of an equal volume (0.4 μL) of saline instead of collagenase. The rectal temperature of the animals was maintained at 37°C throughout the experimental and recovery periods.

### Intracerebroventricular and IP injection

Agomir-183-5p or antagomir-183-5p (0.5 nmol dissolved in 0.4 μL of phosphate-buffered saline [PBS]) was administered before ICH via intracerebroventricular injection. The injection was performed according to an earlier protocol [69]. The mice were anesthetized and placed in a prone position, and a stereotactic head frame was attached. A scalp incision was made along the midline, and a hole was drilled on the right side of the skull (0.5 mm posterior and 1.0 mm lateral to the bregma). Agomir-183-5p or antagomir-183-5p (0.4 μL) was microinjected into the left lateral ventricle through a Hamilton syringe (2.5 mm deep). The needle was kept in situ for a further 5 minutes after injection to prevent possible leakage and then slowly withdrawn over 4 minutes. After the needle was removed, the craniotomies were closed with bone wax.

CoPP and ZnPP were dissolved in 0.2 M NaOH, the pH was adjusted to 7.4, and the solutions were diluted to 1 mg/mL in normal saline. CoPP (5 mg/kg) was intraperitoneally injected 24 hours before collagenase injection and thereafter injected once a day for 4 days. ZnPP (5 mg/kg) was intraperitoneally injected 2 hours after collagenase injection and thereafter injected once a day for 3 days. Needles were inserted into mice in the sham operation group, without injection. tBHQ was dissolved in a solution of 10% dimethyl sulfoxide (DMSO) and 90% corn oil (50 mg/kg) and was intraperitoneally injected at 8-hour intervals, beginning 1 hour after ICH.

### Sequencing analysis of miRNA expression

Low-molecular-weight RNA was isolated from the brains of mice in the ICH 3-day and sham groups using the *mir*Vana RNA Isolation Kit (Thermo Fisher Scientific Inc., Waltham, MA, USA). MiRNA expression profiles were determined with the Illumina HiSeq 4000 platform (Illumina, Inc., San Diego, CA, USA) according to the manufacturer’s instructions. The sequencing process was supported by Annoroad Gene Technology Co., Ltd. (Beijing, China).

### Tissue processing

For immunofluorescence analyses, the mice were subjected to cardiac perfusion with cold saline followed by 4% paraformaldehyde. The brains were removed and fixed in 4% paraformaldehyde at 4°C for 24 hours. After fixation, the brains were embedded in optimal cutting temperature compound (OCT, Sakura Tissue-Tek, Sakura Finetek USA, Inc., Torrance, CA, USA) and coronally sliced into 15-μm sections. For RT-qPCR and western blot analyses, the mice were perfused with cold saline. The brains were then dissected on ice, and the tissues were flash-frozen in liquid nitrogen and stored at –80°C until further use.

### Ferrous deposition analysis

Lillie ferrous staining (G3320, Solarbio Technology Co., Ltd., Beijing, China) was performed to evaluate ferrous deposition. The tissue was fixed in 10% neutral buffered formalin, routinely dehydrated and embedded. The 4-μm-thick sections were routinely dewaxed and rehydrated. The slices were washed with distilled water for 1 minute and then soaked in Lillie stain for 30 minutes before being fully flushed for 2-5 minutes with distilled water. To stain the nuclei, slides were stained with nuclear solid red staining solution (Solarbio) for 5-10 minutes and rinsed with distilled water for 5 seconds. This was followed by conventional dehydration, clearing, and neutral gum mounting. For each mouse, 12 locations were selected (4 fields per section and 3 sections per mouse) to obtain the average number of Lillie-positive cells per mm^2^.

### Lesion volume analysis

To estimate hematoma volumes, the mouse brains were sliced coronally through the needle entry site to obtain serial slices (1-mm thickness) anterior and posterior to the needle entry plane. Photographs of the serial slices were taken, and the lesion areas were analyzed using ImageJ software. Lesion volume (mm^3^) was calculated by multiplying the lesion area in each section by the thickness of the section and adding up this value for all sections containing the lesion [70].

### Brain water content

Brain edema was determined on day 3 by the wet-dry weight ratio method, as described previously [71]. This value was calculated as follows: (wet weight – dry weight)/wet weight of the brain tissue × 100%.

### BBB permeability

BBB permeability was determined 3 days after ICH by EB extravasation [8]. Briefly, EB (2%, 2 mL/kg, Sigma-Aldrich) was injected via the caudal vein 0.5 hours before perfusion. EB leakage was used to assess BBB permeability. The mouse brains were sliced coronally through the needle entry site to obtain serial slices (1-mm thickness) anterior and posterior to the needle entry plane. Photographs of the serial slices were taken, and the EB extravasation areas were analyzed using ImageJ software. The total EB staining volume (mm^3^) was calculated by multiplying the EB staining area in each section by the thickness of the section and adding up this value for all sections with EB staining.

### Neurologic deficits

Six neurologic tests were carried out by an investigator blinded to the treatment groups. These tests assessed body symmetry, gait, climbing behavior, circling behavior, front limb symmetry, and compulsory circling. Performance was scored from 0 to 4 for each test, and the maximum deficit score was 24 [72].

### Cell culture and treatment

The BV2 murine microglial cell line was obtained from Peking Union Medical College (Beijing, China). The cells were cultured in Dulbecco’s Modified Eagle Medium/Nutrient Mixture F-12 (DMEM/F-12) supplemented with 10% fetal bovine serum (Thermo Fisher Scientific), 2 mM glutamine, 100 U/mL penicillin, and 100 mg/mL streptomycin, and incubated at 37°C in a 5% CO_2_ humidified atmosphere. The cells were exposed to hemin (10 μM), agomir-183-5p (50 nM), antagomir-183-5p (50 nM), ZnPP (20 mM), or CoPP (20 mM) for 24 hours. The grouping was similar to that of animal experiments.

### Immunofluorescence

Frozen sections (15-μm thick) or cells that had been fixed with 4% paraformaldehyde were blocked with 10% goat serum at 22°C for 30 minutes and incubated overnight with primary antibodies at 4°C. Goat anti-mouse or anti-rabbit antibodies (1:200) from Jackson ImmunoResearch Laboratories, Inc. (West Grove, PA, USA) were incubated with the sections or cells for 1 hour at 22°C the following day. The following primary antibodies were used: mouse monoclonal anti-HO-1 (1:100, ADI-OSA-110, Enzo Life Sciences, Inc., Farmingdale, NY, USA), rabbit anti-Iba-1 (1:200, ab178846, Abcam Plc., Cambridge, UK), and rabbit anti-myeloperoxidase (MPO, 1:100, ab9535, Abcam). For each mouse, 12 locations were selected (4 fields per section and 3 sections per mouse) to obtain the average number of positive cells. An investigator blinded to the experimental groups analyzed the sections using ImageJ software.

### qPCR

Total RNA was isolated from mouse striatum and BV2 cells sing TRIzol reagent (Beyotime, Shanghai, China) according to a published protocol [73]. A PrimeScript RT Reagent Kit with gDNA Eraser (Beyotime) and a Mir-X miRNA First-Strand Synthesis Kit including primers for U6 (Beyotime) were applied to synthesize cDNA from mRNA and miRNA, respectively. RT-qPCR was performed using an iQ5 real-time PCR system (Bio-Rad Laboratories, Inc., Hercules, CA, USA) with SYBR *Premix Ex Taq* (Beyotime). Every sample was run in triplicate, and the results were analyzed using the 2^-ΔΔCT^ method. U6 and β-actin were used to normalize the miRNA and mRNA levels, respectively. The following primers were synthesized by Tiangen Biotech Co., Ltd. (Beijing, China): HO-1 primers: 5’-CGG GCC AGC AAC AAA GTG-3’ (forward), 5’-AGT GTA AGG ACC CAT CGG AGA A-3’ (reverse); β-actin primers: 5’-TCC TCC CTG GAG AAG AGC TA-3’ (forward), 5’-TCA GGA GGA GCA ATG ATC TTG-3’ (reverse). The first cDNA strands were synthesized from miRNAs using poly(A) tailing.

### Western blot analysis

Briefly, brain tissue or BV2 microglia were homogenized in radioimmunoprecipitation assay buffer (P1003B, Beyotime) containing a protease inhibitor cocktail (P8340, Sigma-Aldrich) and then sonicated on ice. After centrifugation, the supernatant was collected for a western blot assay. Aliquots of each sample, containing 20 μg of protein, were separated by SDS-PAGE and transferred onto a nitrocellulose membrane. The membrane was blocked with 5% nonfat milk in a Tris-buffered saline, 0.1% Tween 20 solution (TBST) for 2 hours (pH 7.4) and incubated overnight with primary antibodies against HO-1 (1:1000, Enzo Life Sciences), Nrf2 (1:1000, ab62353, Abcam), p-Nrf2 (1:5000, ab76026, Abcam), or β-actin (1:1000, ZsBio, Beijing, China), at 4°C. Thereafter, the membrane was incubated with secondary antibody for 1 hour at 22°C, and visualization was performed with a chemiluminescence apparatus (HaiGene, Harbin, China).

### Luciferase reporter assay

The WT HMOX1 3’-untranslated region (3’-UTR), which contains the binding site for miR-183-5p, and the mutant (MUT) HMOX1 3’-UTR were amplified and inserted into the pmiR-RB-REPORT vector (RiboBio Co., Ltd., Guangzhou, Guangdong, China) with XhoI and SacI double digestion. Both recombinant vectors were verified by DNA sequencing. Human embryonic kidney 293 (HEK293) cells were subcultured in 96-well plates and cotransfected with the recombinant vectors, miR-183-5p mimic (miR-183-5p MM), or nontarget control (NC) using Lipofectamine 2000 (Thermo Fisher Scientific). The cells were lysed 48 hours after transfection and subjected to a Dual-Luciferase Reporter Assay (Promega Corporation, Madison, WI, USA) using a Varioskan Flash spectral scanning multimode reader (Thermo Fisher Scientific). *Renilla* luciferase activity was normalized to that of firefly luciferase.

### ELISA

According to the previous grouping, the brain homogenates of mice 3 days after ICH were assessed using ELISA. IL-1β (p1301, Biotime, Shanghai, China), IL-6 (PI326), TNF-α (PT512), and 4-HNE (ab238538, Abcam) levels were determined according to the manufacturers’ instructions. The concentrations of cytokines and 4-HNE (pg/mL) were determined using standard curves obtained from known amounts of IL-1β, IL-6, or TNF-α and were expressed as a percentage relative to their concentrations in the ICH group. BV2 microglia were divided into groups according to the previous experimental design section, the corresponding reagents were added to the cell culture media, and the cells were cultured for 24 hours. At the end of the incubation, the culture media were collected. The concentrations of cytokines and 4-HNE (pg/mL) in the culture media were expressed as a percentage relative to their concentrations in the culture media of cells stimulated with hemin (10 μM).

### Cell viability

BV-2 microglia (2000 cells in 100 μL per well) were seeded into 96-well plates and divided into the following four groups (eight wells per group): the sham group, hemin (10 μM), hemin (10 μM) + agomir-183-5p (50 nM), and hemin (10 μM) + antagomir-183-5p (50 nM). The cells were incubated for 24 hours, after which 10 μL of Cell Counting Kit-8 (CCK-8) (C0041, Biotime, Beijing, China) were added to each well and incubated at 37°C for 4 hours. CCK-8 contains a novel highly water-soluble tetrazolium salt that is reduced by dehydrogenase activity in the cell to form a yellow, water-soluble formazan dye. The amount of formazan dye is directly proportional to the amount of living cells. After incubation, the optical density value at 450 nm was measured using a microplate reader (model 680, Bio-Rad). Cell viability was compared to that in the sham group (100%).

### In situ detection of ROS

### In vivo ROS detection

ROS were evaluated after ICH via in situ detection of oxidized hydroethidine [74]. Hydroethidine (Thermo Fisher Scientific) was dissolved in DMSO and then diluted in PBS to a final concentration of 1 mg/mL. Three hundred milliliters of hydroethidine were injected intraperitoneally on day 3 after ICH, 2 hours before the brains were harvested. Fluorescence intensity of predefined areas of the hemorrhagic striatum in the brain sections was determined. An investigator blinded to the experimental groups analyzed the sections using ImageJ software.

### In vitro ROS detection

A total of 1 mg of hydroethidine was dissolved in 2 mL of DMSO to prepare a stock solution at a concentration of 1.59 mM. The stock solution was diluted to 10 μM in DMEM. After exposure to hemin, agomir-183-5p, or antagomir-183-5p for 24 hours, the above solution was added, followed by incubation for 2 hours, and the fluorescence intensity was determined.

### Statistical analyses

Differences between two groups were compared using the Mann-Whitney *U* test. Statistical comparisons among multiple groups were made using the Kruskal-Wallis test. Statistical significance was defined as *P* < 0.05.

## Supplementary Material

Supplementary Figures

Supplementary Table 1
