# Supplementary material for: miR-183-5p alleviates early injury after intracerebral hemorrhage by inhibiting heme oxygenase-1 expression
Source: Aging (Albany NY). 2020 Jun 29;12(13):12869–95. doi: 10.18632/aging.103343 (PMC7377845; doi:10.18632/aging.103343)
Supplement: Supplementary Figures [file aging-12-103343-s002..pdf]

SUPPLEMENTARY FIGURES

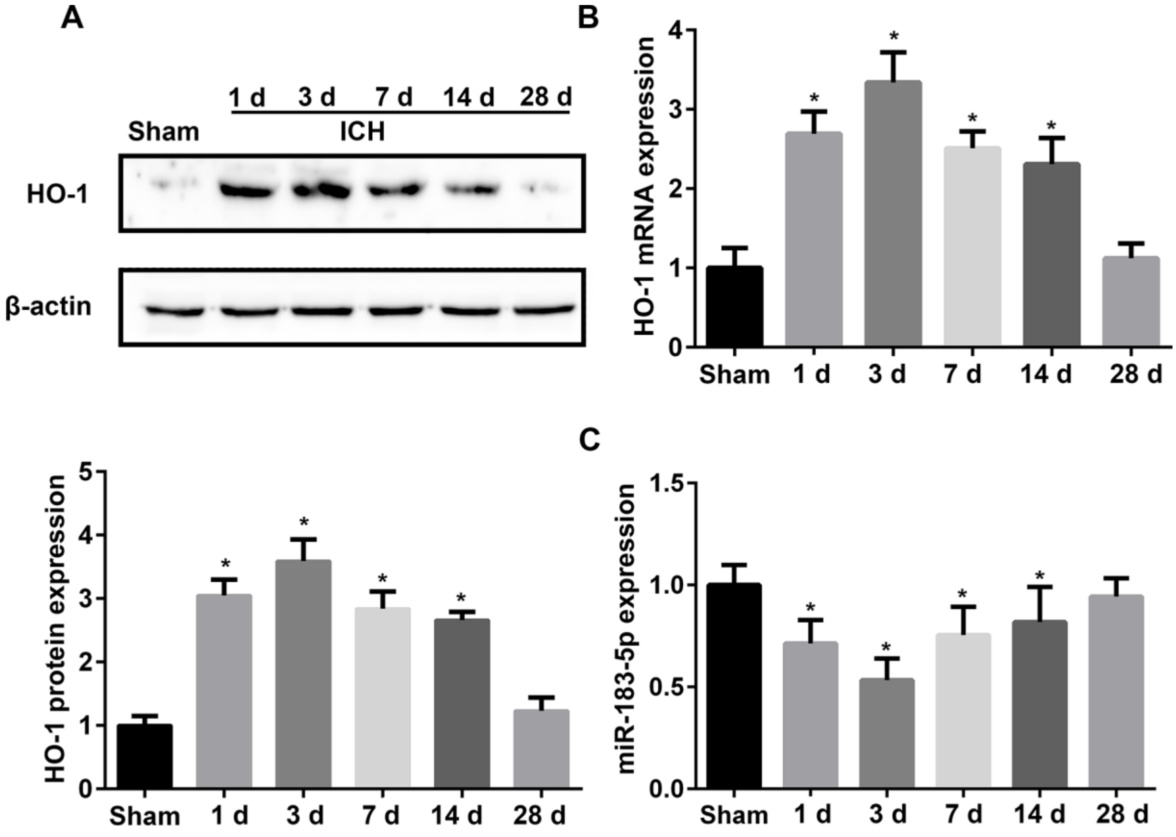

Supplementary Figure 1. A negative correlation was observed between the time course of heme oxygenase-1 (HO-1) expression and miR-183-5p after intracerebral hemorrhaging (ICH). (**A**) Above: western blotting showed HO-1 expression at different time points after ICH. Below: quantitative analysis of HO-1 protein expression at different time points after ICH. n = 8/group. (**B**) Quantitative analysis of HO-1 mRNA expression at different time points after ICH by qPCR. n = 8/group. (**C**) Quantitative analysis of miRNA-183-5p expression at different time points after ICH by qPCR. n = 8/group. Values are presented as the mean ± standard deviation. *P < 0.05 vs. the sham group.

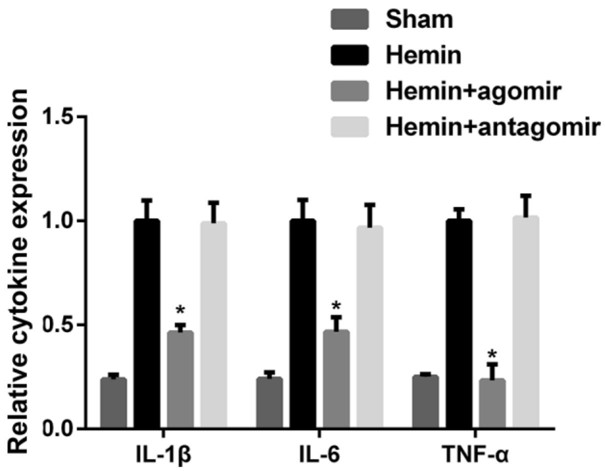

Supplementary Figure 2. Treatment with miR-183-5p reduced cytokine release from BV2 microglia exposed to hemin. Quantitative analysis of cytokine expression in the supernatants of cultured BV2 microglia from different groups at 24 hours after hemin treatment. n = 3/group. Values are presented as the mean ± standard deviation. *$P < 0.05$ vs. the hemin group.

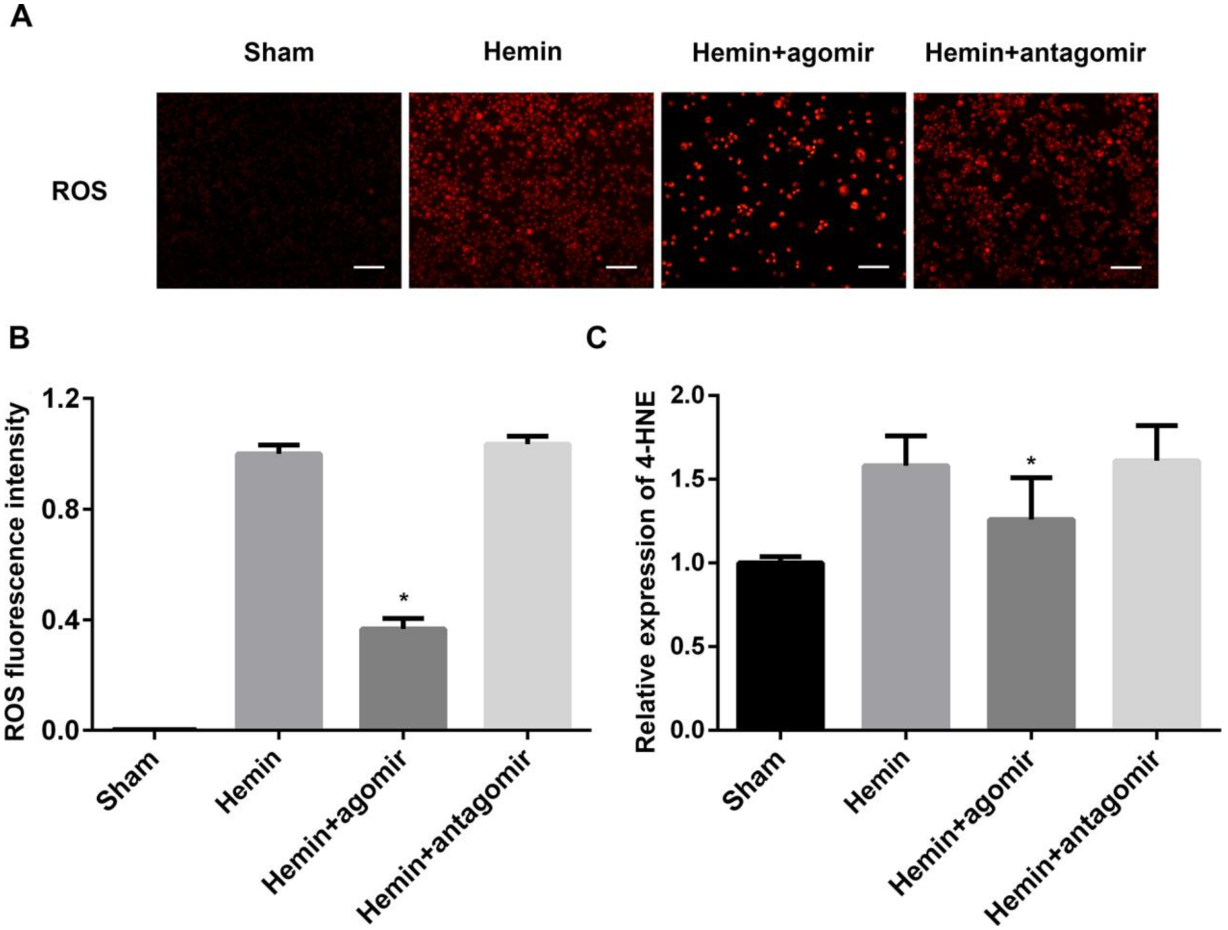

Supplementary Figure 3. Treatment with miR-183-5p reduced reactive oxygen species (ROS) production by damaging BV2 microglia exposed to hemin. (**A**) Representative immunofluorescence images of hydroethidine-positive ROS in BV2 microglia from different groups. n = 3/group. miR-183-5p reduced the number of microglial cells treated with hemin for 24 hours, and the total amount of ROS decreased. (**B**) Quantitative analysis of ROS fluorescence intensity in (**A**). (**C**) Quantitative analysis of 4-HNE in BV2 microglia from different groups. n = 3/group. Values are presented as the mean ± standard deviation. *$P < 0.05$ vs. the hemin group.

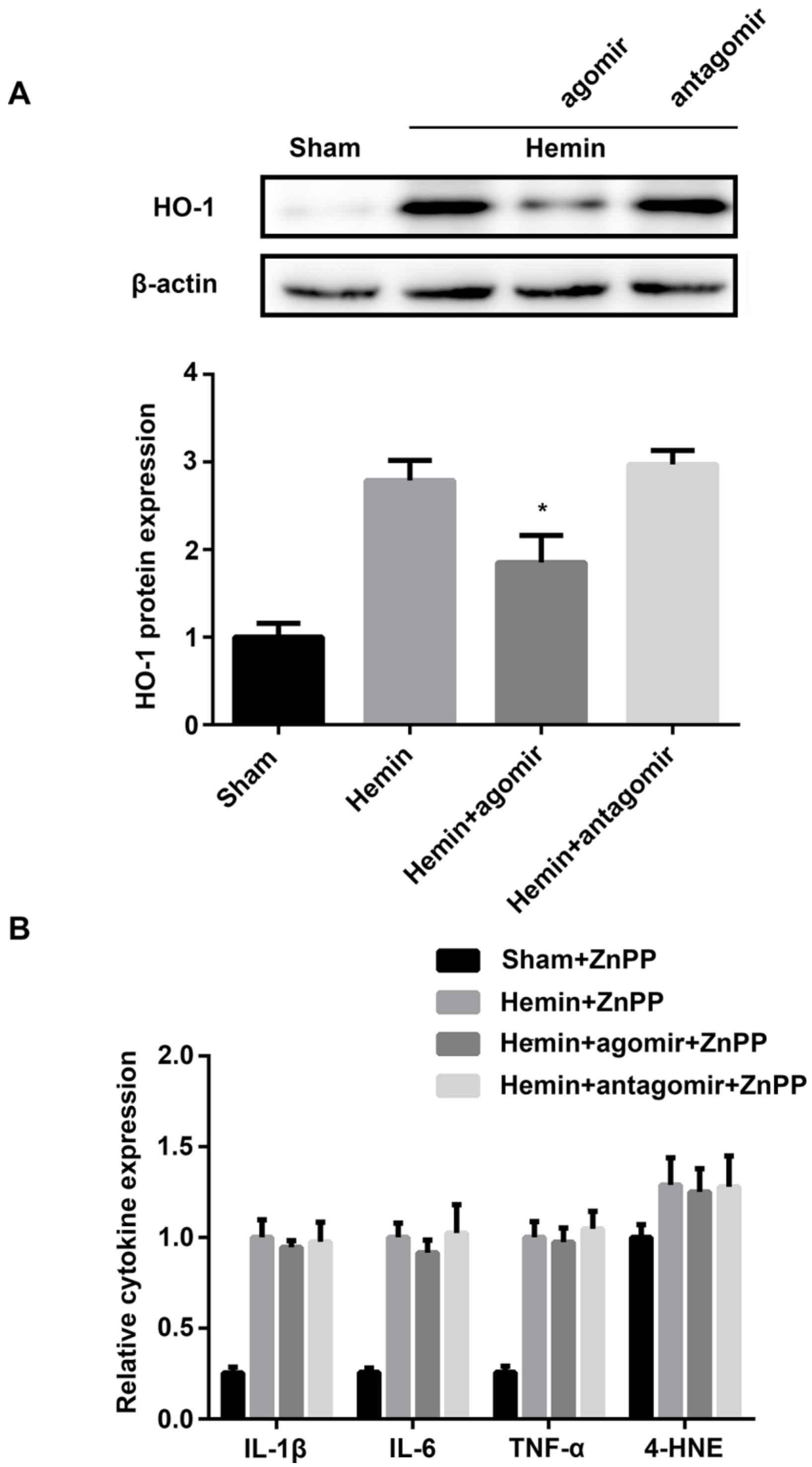

Supplementary Figure 4. miR-183-5p alleviated inflammation and oxidative damage by directly targeting heme oxygenase-1 (HO-1). (**A**) Above: western blotting revealed that miRNA-183-5p downregulated HO-1 expression. Below: quantitative analysis of HO-1 protein expression in different groups. n = 3/group. (**B**) Quantitative analysis of cytokines from the supernatants of cultured BV2 microglia and 4-HNE expression in BV2 microglia from different groups pretreated with the HO-1 inhibitor zinc protoporphyrin IX (ZnPP) at 24 hours after hemin treatment. n = 3/group. Values are presented as the mean ± standard deviation. $^*P < 0.05$ vs. the hemin group.

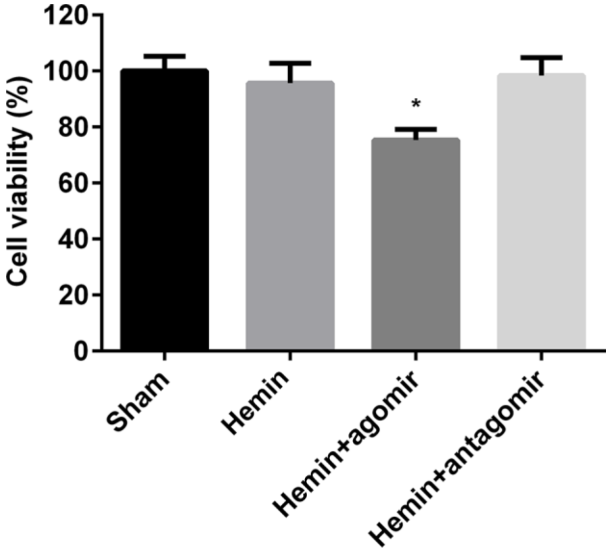

Supplementary Figure 5. miR-183-5p reduced the viability of BV2 microglia exposed to hemin. The Cell Counting Kit-8 (CCK-8) assay revealed that administration of agomir-183-5p reduced the optical density values, as determined using a microplate reader. n = 3/group. Values are presented as the mean ± standard deviation. *$P < 0.05$ vs. the hemin group.

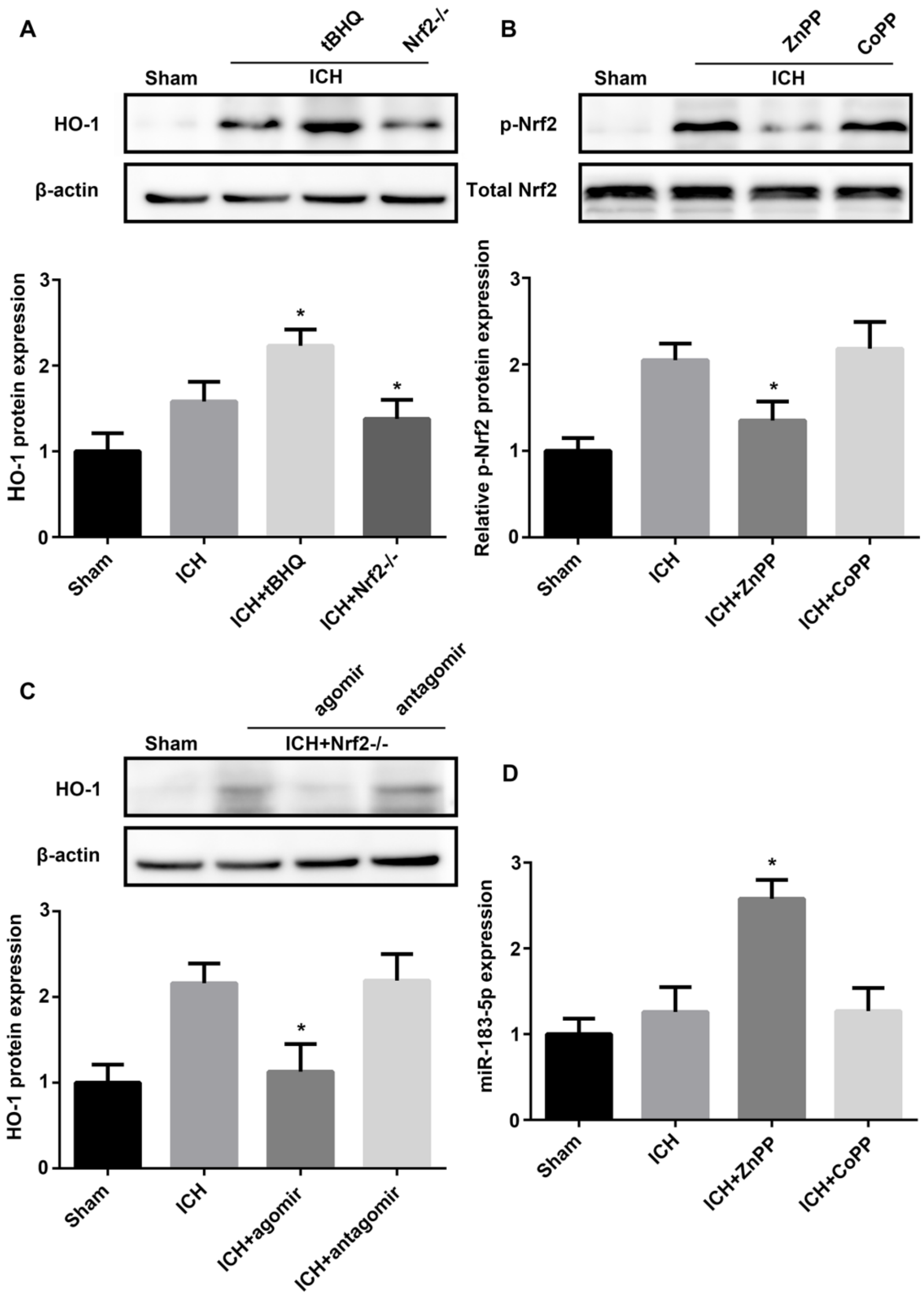

Supplementary Figure 6. Regulatory relationship between miR-183-5p, heme oxygenase-1 (HO-1), and Nrf2. (**A**) Above: western blotting revealed that Nrf2 activation promoted HO-1 expression in mice at 3 days after intracerebral hemorrhaging (ICH). n = 8/group. Below: quantitative analysis of HO-1 protein expression in the different groups above. (**B**) Above: western blotting revealed that HO-1 promoted Nrf2 activation in mice at 3 days after ICH. n = 8/group. Below: quantitative analysis of relative p-Nrf2 protein expression in the different groups above. (**C**) Above: western blotting revealed that miR-183-5p reduced HO-1 expression independent of Nrf2 in mice at 3 days after ICH. n = 8/group. Below: quantitative analysis of HO-1 protein expression in the different groups described above. (**D**) RT-qPCR revealed that miR-183-5p expression was increased in the presence of the HO-1 inhibitor zinc protoporphyrin IX (ZnPP) at 3 days after ICH. n = 8/group. Values are presented as the mean ± standard deviation. *$P < 0.05$ vs. the ICH group. CoPP, cobalt protoporphyrin IX; tBHQ, tert-butylhydroquinone.

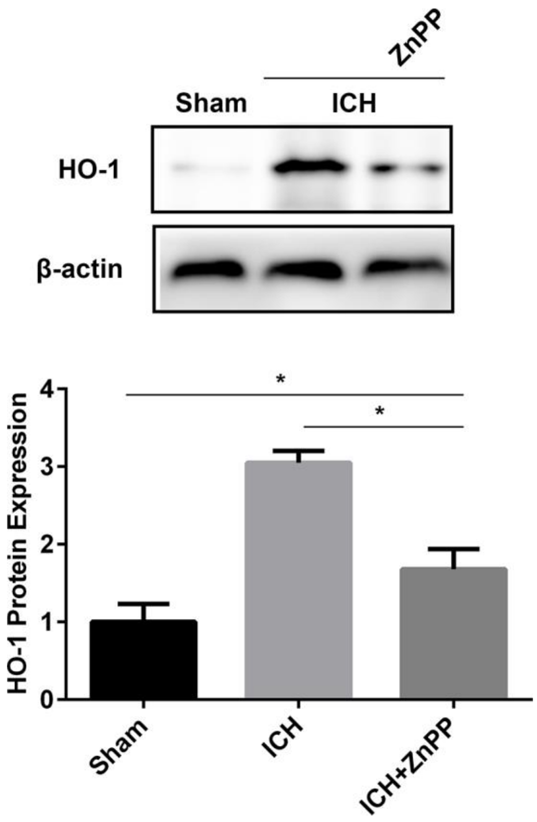

Supplementary Figure 7. Intraperitoneal injection of ZnPP inhibited the expression of HO-1 in brain tissue of mice 3 days after intracerebral hemorrhage. n = 8/group. Values are presented as the mean ± standard deviation. *P < 0.05. ZnPP, zinc protoporphyrin IX
