# Supplementary material for: miR-183-5p alleviates early injury after intracerebral hemorrhage by inhibiting heme oxygenase-1 expression
Source: Aging (Albany NY). 2020 Jun 29;12(13):12869–95. doi: 10.18632/aging.103343 (PMC7377845; doi:10.18632/aging.103343)
Supplement: Supplementary Table 1 [file aging-12-103343-s001..docx]

**Supplementary Table 1. mRNAs that may be involved in the ICH process and bind with miR-183-5p.**

| Putative targets of miR-183-5p after ICH | Differentially expressed mRNAs after ICH | mRNAs that may be involved in the ICH process and bind with miR-183-5p |
| --- | --- | --- |
| Hk2  Stk38l  Capns1  Grik3  Foxn1  Hltf  Rgs20  Ppp5c  Abca13  Hmox1  Pcyt1a  Napa  Apbb3  Fzd3  Dqx1  Tmem8c  Ppp2cb  Adamts13  Efr3a  Pom121l2  Ncor1  Unc45b  Pank3  Lhx9  Sema6a  Kitl  Tmcc3  Slc5a8  Rnf185  Mtif2  Cenpo  Mrc2  Helz  aminoacylase  Ankfy1  Serpina3n  Cdc42bpb  Slc6a3  Rab3c  Bmpr1a  Opn4  Dcp1a  Npr3  Zscan26  Trio  Rnf19a  Csmd3  Drosophila  Cacna1i  Atp13a3  FRAGMENT  Prkdc  Birc6  Pja2  Qpct  St6gal2  Kdm4b  TBP  Dnajc18  Lama3  Lpxn  Kcnk4  Tcf7l2  monocarboxylic acid transporters  Pi4k2a  Drosophila  Idh1  Lancl1  FRAGMENT  Rqcd1  aminopeptidase B  Ccdc93  Cacna1s  Nfasc  Adck3  Kif26b  Cenpf  Ush2a  Neb  Lrp2  Spred1  Kif3b  Ntsr1  Ralgapb  Sucnr1  Nbea  Veph1  Notch2  Fam198b  Ash1l  Asph  DHHB-MTASE  Ptbp3  Zdhhc21  FRAGMENT  Cpt2  Csf3r  Ufd2  Mfn2  Cd38  Pgm1  Flt1  Cyp3a13  Trim24  Fam131b  1700074P13Rik  Tacr1  Cntn3  Cand2  Rasgrp4  F8  Atrx  Flna  Arhgap6  Mak16  Sin3b  Adgrg1  Jak3  Has3  Abcc10  Foxj3  Myh2  Ttf2  Map2k4  Clasp2  Idua  Tmem35  Asb13  activator 1  Ptgdr2  Lrrc58  Med13  Gpatch8  Fam171a2  Tjp3  Foxn2  Tbr1  Neu3  S. cerevisiae  Syt1  Ppp1r11  Btg1  Cdh8  Cfap221  Jade3  Tecta  Rara  Arap2  Mical2  Sesn1  Plcl1  Tmem255b  Parp12  Ric1  Wdr26  Bpgm  Hrh3  Zfp750  Foxk2  9130011E15Rik  Olfr906  Plcb2  Gbp7  Pitpnm3  Dlk1  Erf  Arhgef10l  Hectd2  Katnal1  Obox6  Pramel1  Ammecr1l  Klhl23  Ptchd4  Tmem183a  Arel1  Sin3a  Fam199x  Stk40  Hfm1  Fam160a2  Fzd5  Pigw  Asxl3  Tmem86b  Lhfpl2  Serpinb9  C2cd2  B430212C06Rik  Olfr914  Slfnl1  Rell1  Tnrc6b  Kcna3  Nwd1  Phldb1  Lrp1b  Rarres1  Aff4  Armcx4  Arhgap23  Olfr921  Fam171a1  Slc6a7  Olfr901  Olfr352  1,4-BETA-N-ACETYLMURAMIDASE  Fate1  Unc80  Myh8  Ero1lb  E030024N20Rik  Olfr66  Olfr25  Fragment  Vwa8  Olfr913  Hydin  Ctnna3  Akap6  Tgif2  Dgkk  Kcnj15  Olfr911-ps1  Ipcef1  Med14  Mir101b  Serpina3f  Vmn2r37  Adgrv1  Znf41-ps  Vmn2r42  Gm5499  Csf2rb2  Myh7b  B3galt5  Yae1d1  Lrrc55  Emc1  Cela3a  1700024P16Rik  Hoxd3  Gm6071  Vmn2r-ps2  Gm11841  Gm13050  Gm14006  Gm16421  Setd3  Gm14153  Gm11839  Gm12842  Gm5883  Gm14534  Xlr3a  Gm13400  Gm11251  Gm12295  Gm12381  Gm14273  Gm14204  A230108P19Rik  1500017E21Rik  Gm13493  Vmn2r94  Vmn2r42  Gcnt4  Vmn1r-ps61  Gm5117  Olfr893  Olfr904  Olfr143  Olfr907  Vmn1r77  Gm5558  Gm18336  4933408J17Rik  Gm5205  Gm7063  Gm20954  Gm5835  E330023G01Rik  Gm6075  Gm36569  Snrpn  Gm6074  Igh  Lypla1  C57BL/6J Female  Gm18555  Gbp7  Gm9523  Gm5981  Teddm3  Gm19078  UHG  Gm6063 | Lair1  Akr1b8  Cpt2  Galnt12  Olfml3  3010003L21Rik  Mvp  Mx1  Ercc6l  BC030867  Zmynd15  Efcab11  Adm  Neil3  1700049L16Rik  Stat5a  BC055324  Fgfrl1  Pmp22  Il21r  Il1rap  Col16a1  Tgfb1  Gm10693  Cchcr1  Rhoc  C3  Afp  C1qb  Mgmt  Cst7  Dennd1c  Trip13  Sgol1  Fam64a  Cdca5  Snap23  Cd59b  Tmem37  8430408G22Rik  Ms4a14  Clec7a  Cklf  LOC102640804  Mlph  AA467197  Mtus1  Pctp  4632428N05Rik  Meis1  Ifngr1  Oas1a  P2ry10  Bub1  Fam26f  Gusb  Trim30d  Rac2  Plce1  Slc16a3  Gsdmd  Mapkapk3  Cd84  Cdca7l  Pm20d1  Lcp1  Plxna4os1  Ptx3  Irx1  Gimap1  Serp1  Cflar  Bin2  Csf1  Plbd1  Tbc1d4  Lyl1  Nek6  Ms4a4a  Gm5150  Gng5  Ltc4s  Rad51  Tnf  Mcm7  Lpar6  Fam101a  Mcm4  Cdca8  P2ry6  Tmpo  Ccr1  P2rx7  Cd300lb  Igfbp2  Fam227b  Anxa5  Batf  Igf2bp2  Rrm2  Plk1  Hmox1  Wdfy1  Lmnb1  F13a1  Gm5127  Gm2990  Ly96  Mgarp  Dok1  Car5b  Havcr2  Samsn1  Birc5  Gmnn  Slc14a1  Galnt15  Ulbp1  Ncf2  Hbegf  Gfap  Cyba  Synpo  Myl12a  Slc25a21  Mb21d1  Dennd2d  Tspan4  Stat3  Stab1  Smad5  Gngt2  Laptm5  Ube2c  Itgb5  Cdc6  Stard5  Oas1a  Susd3  C1qc  Hist1h2an  Cutal  Ccnb2  Tnfrsf1a  Grn  Zfp36  Glycam1  Xdh  Mns1  Cdkn2a  Tax1bp3  Gpr65  Sirpb1b  Liph  Spic  Spata6  Nckap1l  Themis2  Slc6a19  Cmtm7  Cd109  Asf1b  Ttk  Laptm5  Ppp3cc  Fcna  Bcl3  Hcst  Lamp2  Gm14625  Lrrfip1  Tacc3  Nde1  Ccl2  Clec4n  Glipr1  1190002F15Rik  Hk2  Cd180  Csf2rb  Gm4907  H2-Aa  Esyt1  Klk6  Hspb6  Smc4  Gm5593  Fcgr2b  Kif18b  Nox4  Sulf1  Naalad2  Mob1a  Aif1  Hist1h2bq  Gldn  Tec  Asap3  Cd52  Gm20746  Gpx1  Pole  Pdlim5  Gltp  Gm5150  Ccnb1  Emp3  Slco2b1  C5ar2  Mcm2  Gm4610  Ripk1  Trem2  Rnaset2a  Mmp3  Nat2  Nusap1  Gfap  Hist1h2ab  Tcirg1  Eme1  Ttc12  Slc16a10  Slc29a3  H2-Q7  Gimap4  Tgm1  Prelid2  Knstrn  Tyms-ps  Cxcl16  Plin2  Rbm47  Col4a1  Gp49a  Apobr  Steap4  Smarcd2  Spint1  4930519N06Rik  Slc25a13  Nedd1  Ftl1  Myd88  Clhc1  Prss46  Xlr  Gm10684  Ermard  H2-K1  Cenpp  Tmem140  Ccl6  Tor3a  Slc7a7  Pycard  Dclre1b  Cks1b  Tfec  Slfn2  C5ar2  Dpep2  Syk  Gm5637  Osmr  Mr1  Pqlc3  Rab27a  1810011H11Rik  Rps6ka1  Dapp1  Ttc12  Tmem252  Pik3cg  Lpxn  Capg  Trim12c  Parp3  Clec1b  Tnfaip8  Ugt1a6b  Lgals9  Pik3cg  Lyn  Cntf  Il10ra  Csf1r  Gm14005  Syngr1  Myo1f  Pkn3  Cdk5rap2  3830403N18Rik  Lilrb4  Gdf3  Arhgef39  Ada  Espl1  Arhgap9  Rab7l1  Rnf135  Apoc1  Csprs  Lcn2  Ddx11  Ctss  Slamf6  Plcd4  Kntc1  Apobec1  Fabp4  Esco2  Kif20a  Ube2t  Gm10487  Msr1  Ms4a4c  Mtr  Tnfsf9  Cenpk  Slc25a45  Hk3  Lgals1  LOC102640775  Cflar  Tnfrsf13b  Birc5  Cdkn2c  Kif2c  Pla2g4a  Ptgr1  Krt85  Mdfi  Lgals3bp  4632434I11Rik  9630013D21Rik  Mcm3  Bcl6b  Mcm10  Dera  Cenpl  Tnfsf8  Gins1  Lpl  Pydc3  Pter  P2ry13  Runx1  Gen1  Ucp2  Igfbp2  Ms4a6b  D8Ertd158e  Vwa5a  B2m  Cttnbp2nl  Sgms2  Gipc2  Fignl1  Lrp1  Tlr7  Cxcl1  Phf11d  Blnk  Slc2a4  Cpsf4l  Mefv  Trim30b  Klrd1  Cpt1a  C130050O18Rik  F11r  Hn1l  Cd14  Dhrs4  Sirpb1b  B430306N03Rik  D730045A05Rik  Krtap11-1  Prokr1  Itga6  Sqrdl  Marveld3  Pip5k1a  Mcm3  Pvrl2  Ehd4  Serpinf2  Fermt3  BC100530  Tax1bp3  Nxpe5  Bub1b  Tmem176b  H2-D1  Timp2  Pla1a  E2f8  Lrmp  Hspb8  1700023L04Rik  Gins2  2810442I21Rik  Prdx6  Dbf4  Cxcr4  Ncf2  Prdx6  Fam25c  Asph  Mtbp  A2m  Disc1  Mmp8  1700023E05Rik  Gm10872  Tagln2  Itgax  Ezh2  Mrc1  Pirb  Mgst1  Slc16a10  2810417H13Rik  Trim47  Bmp2k  B4galt1  Ccnb1  Cks2  Renbp  Raet1e  Rab7l1  Nes  Bora  Gpr160  Gm5086  Ap1s2  1700112E06Rik  Cdc25c  Cd72  Irf9  Slc43a1  Poln  Nod2  Cx3cr1  Aff1  Adap2  Ncf4  Tgfbr2  Ip6k3  Mcoln3  Gm2030  Gm5934  Pik3r5  Slc11a1  Dlgap5  Sele  S100a11  Thbs1  Ncaph  Efemp2  Eif2ak2  Tnfaip2  Cd300lf  Spc25  Cebpa  P4hb  Hmgb2  2610020H08Rik  Sirpb1a  Cxcl10  Hmha1  Rasa4  Epha2  Adam3  Ifi204  Emp1  Anxa3  Gpr114  Csf1  Cd38  Syngr2  Angpt2  Olfr920  Pira7  Trim12c  Gal3st4  4930427A07Rik  Cenpl  Sp140  Srgn  Tlr2  Rbm47  Serpina3n  Gpr183  Psors1c2  Mmp2  Serpinb5  Mki67  Abcc3  Ms4a4b  Hoxb4  Ifitm1  Rgs1  Fgl1  Alox5  Inadl  5430427O19Rik  Slc16a6  Mmp13  4930556M19Rik  Gltp  Fcgr4  Pdpn  Ly86  Tlr4  Ifit3  Arhgap30  Rab13  Gcnt1  Spint1  Fcgr3  Lcp2  Rgs21  Ifi203  Baz1a  Pdlim4  Maff  Ahnak  Casp4  Cfh  Cd48  Fam129a  BC026585  Slc30a7  Gm6583  Ncapg2  Gm13272  Lrrc25  Cenph  Ddx60  Usp18  Nsl1  9530051G07Rik  Cybb  Gpr35  Vps13c  Pld4  Gxylt1  Galnt12  Lifr  Hpgd  A530064D06Rik  Jam2  Xlr  Slamf7  Nt5dc2  LOC102641688  Cd200r4  Tgif1  H2-K2  Alox5ap  Sh3bp2  Glipr2  Gm5547  Cdca8  4930579G24Rik  Ecscr  Clic1  Dna2  Tec  4632428N05Rik  Lat2  Il4ra  Tmem154  Tmed10  Oas1f  Plscr1  Arhgap19  Kcnk6  Golm1  Kif4  Hist1h2ah  Cfhr2  Kif11  Slfn4  Aurka  Neat1  Sh2d1b1  Cfh  Ccl24  Map3k8  Pygl  Arhgef15  Slc35d2  Sp110  Trim30a  A630033H20Rik  Cebpd  Rab3il1  Pde3b  Skap2  Chtf18  Prc1  Igfbp7  Tnfrsf26  Casp8  Oit3  Padi4  E030003E18Rik  Fes  Kif23  Mdfic  Lilrb4  Slc31a2  2210406H18Rik  Acer2  Clspn  Flnc  Nqo2  Igf1  Cyp4f16  Il6  Knstrn  Cd1d1  Fes  Aurkb  Spata6  Cpne3  Cenpk  Kdelr3  Cdt1  Dock8  Fhod1  A130071D04Rik  Apobec3  Mcm6  Agbl2  5430435G22Rik  Chrdl1  Slc37a2  Parpbp  P2ry2  Ccl11  AA667203  Inpp5d  Anxa4  Cx3cr1  4933440N22Rik  Tmem173  Bmp2k  Spc24  Itga5  Sirpb1a  Traf3ip3  Ccl12  Stag3  Lgals3bp  Gpr84  Mmp19  Trpm3  Vav1  C4b  Cdc6  Orc1  1700001K23Rik  Ahnak  Oasl1  Gdf15  Gmip  Palld  Il1a  Ticam2  Brca1  Cln5  Ormdl2  Nuf2  Tlr6  Ppap2c  Fas  Lrr1  Cd44  Wipf1  Tlr1  Fgl2  4930486L24Rik  Mki67  BC028528  Npl  Ifi30  LOC545966  Lcp1  Col5a3  A330015K06Rik  Ska1  Cd200r1  Cnn3  Mnda  Zcwpw1  Nox4  Adcy7  Ncapg  Wdfy4  Tnfrsf23  S1pr2  6330407A03Rik  Cxcl5  Galm  Ddx21  Psmb8  Gpsm3  Btk  Nfam1  Gm5532  Akr1c13  Lpin3  Slc43a3  Aldh1l2  Fam227b  Hck  Cdk1  Loxl2  AI607873  Rbl1  Tubb6  Spp1  Fxyd1  Prdx6  Myc  Casc5  Rrbp1  Itgb1  Ska3  Ugt1a10  Osgin1  Bst1  Ddx58  Cenpe  Ctsh  Fam83d  Angptl4  Mmp27  Cd33  Dock2  Ccnb1  Gsap  Prune2  Emr1  C530044C16Rik  Cd48  Ascc2  Treml2  8030462N17Rik  Snx20  Cd86  Tpx2  4930427A07Rik  Micall2  Cmtm2a  Tns3  Ldlrap1  Itgb1  Mcm5  Rnase4  Map4k4  Spi1  Cd63  Tnfrsf18  Hrsp12  Cd37  Birc3  Gm5168  Zc3hav1  Tor4a  Nrros  Plk4  Cela1  Tuba1c  Ccr5  Dhh  Vim  Tbxas1  Trpv4  Bcl2a1c  Hist1h1b  Il2rg  Il13ra1  Cdk2  Pyhin1  Tagln  Myo1g  Il15  Ctsz  Anxa2  Ms4a6d  Cd9  Myo1f  Arhgdib  Gtse1  Gm1993  Fyb  Nhlrc3  Dscc1  Psma8  Ckap2l  Cdkn3  Slpi  Fam46c  Slamf9  Rsad2  Dyrk4  Fli1  Ntrk2  Pram1  Cysltr1  Lilra6  Gpr34  S100a16  Reep4  Naip1  Pon3  Rom1  Hist1h2ag  Tnfrsf1b  Plaur  Hcls1  Pdpn  Gm16340  Cep72  Gm2030  Igfbp3  Sgol2  Adam8  E2f2  Cdk1  5031414D18Rik  Fblim1  Hist1h4i  Aspg  AB124611  Il1r2  Cks1b  1810034E14Rik  Il10rb  Arhgap11a  Hvcn1  Cyth4  Mdfic  Tfpi2  Pf4  H2-K2  Gm8995  Ftl1  Smim3  Irak4  Clca2  Npc2  Ptprc  Hist1h1c  Ctla2b  Tgm2  Serpine1  Vwf  Sdc1  Acp5  Adora3  Ltbr  Cdc20  Btc  Gch1  Ch25h  Tnfrsf12a  Vangl1  Gatsl3  Igsf6  Stfa2l1  Cym  LOC102633627  Rad51ap1  Arg1  Lrrfip1  Ddx43  Pira11  Lipa  Csf1  Rbl1  Csf1r  Hfe  Mir17hg  Trim16  Gas2l3  Nsl1  Vav1  Pter  Arpc1b  Troap  Ehd4  Cdca3  Matn3  Fn1  Il2rb  Iqgap3  Renbp  Was  Flna  E2f7  Phox2a  Vsig4  Hhex  Slfn10-ps  Duoxa1  Carhsp1  Trim34a  Tgfbi  B3gnt5  Srebf2  Tspo  I830077J02Rik  Arhgap11a  Ecm1  Hmga2  Gpnmb  Fbxo5  Ptpn7  G6pd2  Sash3  Folr2  Dtx3l  Racgap1  S100a4  Gmfg  Slco1a5  Nek2  Slc6a11  Trim34b  Arhgap11a  Parva  Galnt6  Gm11545  Ptpn6  Ms4a7  Foxm1  Dap  Nras  Hist1h2ao  Gm14625  Hpgds  Mab21l3  Mfsd7a  Eps15l1  Pilra  Htr2b  Tes  Parvg  Ube2c  Mpeg1  Lonrf3  H2-Ab1  Pbk  Skor1  9630023C09Rik  Tnfaip8  Unc93b1  Cenpf  LOC102638793  Lcp2  Pilra  Tgfbr2  Pyhin1  Apod  Rbms1  A630001G21Rik  Serpina3i  Gpam  Ctsd  Gm8675  Cep128  AI429363  Serhl  Cpt2  Fosl1  Lacc1  Dlgap5  Ifi27l2a  Vwa5a  Fcgr1  Cd36  Mt1  Socs3  Tcn2  Ccl3  Klhl6  Cenpa  Ckap2  Sparc  Emilin2  Cd68  Fanca  Ly9  Metrn  Kifc1  Abca1  Traf3ip2  Stat3  Ikzf1  Gcnt1  Kif23  Nmnat3  4933432I03Rik  Cmtm2a  Plcg2  Abhd15  Rbm3  Tpx2  Sbno2  Upk3bl  Tlr13  Msn  H2-DMb1  S100a6  Sdf2l1  B3gnt5  Lgals3  Exo1  Ctsl  Camk2d  Fam111a  Fignl1  Atf7  Sparc  Naip2  Pla2g5  Aim2  Fcer1g  C1qa  Stx11  Uhrf1  Gm4297  Creg1  Card9  Msr1  Tmem176b  Timp1  Cybrd1  Nfatc1  Slco2b1  AU022793  Plscr2  Pyroxd2  Sdc4  Spata1  Ttf2  Nid2  Mastl  Csf2rb2  Brip1  Slc40a1  9330175E14Rik  Rcbtb2  Lhfpl2  Tmem52  5730416F02Rik  Fam89a  Ccnd1  AI467606  Tm4sf1  Rhoj  C5ar1  Hexb  Susd3  Spidr  Chaf1a  Trim30e-ps1  Itpripl2  Adamts1  Tram1  Ampd3  Chek2  Tmem220  Cdca2  Birc5  Spata13  Ifi204  Tmem194  Tnfaip2  Fmo2  Epx  Stt3a  H2-T10  LOC102639543  1700009J07Rik  Gm9999  Gm16428  Irf8  Gm9895  Ctsc  Ermard  Nanog  Lrig1  Cytip  Tpm4  Slfn3  Mybl2  Cep55  Abi3  Frmd8  Bag3  Tmod3  Tnfsf13b  Heatr5a  Pros1  Slc10a6  Tnpo1  Pbxip1  LOC102639543  Asb10  A430104N18Rik  Ptafr  Cnn3  Zwilch  Ctsd  Hcar2  Sgpl1  Cnn3  Vwf  Gpx8  Aspm  Slc1a5  Pilra  Cysltr1  Apod  Gm5414  Vamp8  Pyhin1  Pald1  Cenpi  Zwilch  H2-T3  Ccdc69  Sbno2  Fabp7  Irak3  Slx  Tagln2  Dhx58  Cd93  Plau  Efcab4b  Ankle1  Polh  Dbi  Ect2  Mboat1  C330027C09Rik  Cenpn  Ddc  Kif18a  Ccl5  Nde1  Serpina3i  Sla  Ikzf1  Tmpo  Evi2a-evi2b  Sirpb1a  Clec4e  Cx3cr1  Ccl9  Arl11  Itgb2  Casp4  Tor3a  Cd1d1  Olfml3  Hapln2  Mxd3  Fcrls  Npm1  Clec4a3  Mcl1  Gm9999  Hvcn1  Kif2c  Clec4d  Ccna2  Diap3  Cd79b  Slc16a9  Frrs1  H2-Ab1  Mybl1  Parp9  Hells  4930469K13Rik  Ccdc18  Depdc1a  Bard1  Gm8013  Zc3h12d  Tlr7  Cmtm3  AI317395  Saa3  Ms4a4c  Il1r1  Igf1  Sh3tc1  Gfap  Galnt4  Acads  Ccr2  Lgmn  Hn1l  Fyb  Rhoc  Hmox1  Apbb1ip  Hes3  Tyrobp  Pnp  2610203C22Rik  Cd300lf  Gm5068  Bst2  Lrrk1  Melk  Ifi35  F7  Pole2  Mrps6  Tuba1c  Lrr1  Shmt1  Wipf1  AF251705  Emr1  Rad54l  Clec1b  Zc3h12a  Pbxip1  Ptbp3  Hexa  Mfsd1  Tnni2  Arl5c  Pbxip1  Rtp4  Pira2  Rassf4  Kpna2  Sh3glb1  Fut4  Hist1h3g  1300002K09Rik  Insl6  Ang3  Lrrk1  Tln1  Ms4a6c  Ugt1a6a  Cyba  Skor1  Phldb3  Tmem106a  Sp140  Ccnf  Btk  Kif15  Rhbdf2  Epsti1  Tmem176a  Hsd3b7  Phf11b  Dnase1l1  Acp5  Ostf1  Sult6b1  Ppic  Kcnn4  Mafb  Nek6  BC055324  AI607873  Sucnr1  Top2a  Mmp12  Plek  Tlcd2  Bcl2a1d  Procr  Cyp1b1  Runx1  Has2  Ccl7  P2rx7  Plscr1  Chst2  5033418A18Rik  Neurl3  Slc16a3  F10  Apln  Fgl1  Plekhg2  Ggta1  Ifitm1  Col2a1  Rab32  Chil1  Gpr137b  Dhrs1  Asb7  Shcbp1  Slc4a11  Rnf213  Phf11d  Tmem119  Slamf8  Chaf1b  Prdx1  Rps27l  Hpgd  Mapk1ip1l  Trf  Gm14548  Sft2d2  Dab2  Gsg2  Ttc12  Lyz1  Ncf1  Creb3l2  Cp  Ier3  Fanci  Rab20  Tep1  Mis18bp1  Ptger4  I830012O16Rik  Trf  Cpne3  Gsg1  2310075C17Rik  Creg1  Ctla2a  Gmnn  Scarb2  Wfdc17  Grap  Ecm1  Dnaic2  Ppbp  2200002D01Rik  Decr1  Creg1  AI427809  Rtkn2  Pstpip1  Ticrr  C3ar1  Prdx6  9330188P03Rik  Kif20b  Igf2bp2  Oip5  Cast  Ifih1  Phf11a  Tmco4  Cotl1  Dennd2c  Mlec  C1s1  Lamp2  Icam1  Parp14  S100a4  Gm5431  Slc37a2  Upp1  Atf3  Ptpn18  Cd72  G630090E17Rik  Ece2  Arhgap18  Ptplad2  Smagp  Lpl  Ucp3  Xrcc2  Akr1c12  Rrm1  Litaf  Tk1  Slc15a3  Nt5dc1  Haus4  Mt2  Lyz2  Ripk3  Smpdl3b  Naip6  Irf5  Elk3  Cd68  Bst1  Il7r  I830012O16Rik  Mgst1 | Hk2  Hmox1  Serpina3n  Lpxn  Cenpf  Sucnr1  Asph  Ptbp3  Cpt2  Cd38  Flna  Ttf2  Lhfpl2  Csf2rb2 |
